# Blockchain assisted signature and certificate based protocol for efficient data protection and transaction management in smart grids

**DOI:** 10.1371/journal.pone.0318182

**Published:** 2025-05-28

**Authors:** Keyan Abdul-Aziz Mutlaq, Vincent Omollo Nyangaresi, Mohd Adib Omar, Zaid Ameen Abduljabbar, Junchao Ma, Mustafa A. Al Sibahee, Abdulla J. Y. Aldarwish, Ali Hasan Ali

**Affiliations:** 1 School of Computer Sciences, Universiti Sains Malaysia, USM, Gelugor, Penang, Malaysia; 2 IT and Communications Center, University of Basrah, Basrah, Iraq; 3 Department of Computer Science and Software Engineering, Jaramogi Oginga Odinga University of Science & Technology, Bondo, Kenya; 4 Department of Applied Electronics, Saveetha School of Engineering, SIMATS, Chennai, Tamil Nadu, India; 5 Department of Computer Science, College of Education for Pure Sciences, University of Basrah, Basrah, Iraq; 6 College of Big Data and Internet, Shenzhen Technology University, Shenzhen, China; 7 Shenzhen Institute, Huazhong University of Science and Technology, Shenzhen, China; 8 Department of Business Management, Al-Imam University College, Balad, Iraq; 9 Department of Management and Marketing, College of Industrial Management for Oil and Gas, Basrah University for Oil and Gas, Basrah, Iraq; 10 National Engineering Laboratory for Big Data System Computing Technology, Shenzhen University, Shenzhen, China; 11 Department of Mathematics, College of Education for Pure Sciences, University of Basrah, Basrah, Iraq; 12 Technical Engineering College, Al-Ayen University, Dhi Qar, Iraq; Xiamen University Malaysia, MALAYSIA

## Abstract

Smart grids collect real-time power consumption reports that are then forwarded to the utility service providers over the public communication channels. Compared with the traditional power grids, smart grids integrate information and communication technologies, cyber physical systems, power generation and distribution domains to enhance flexibility, efficiency, transparency and reliability of the electric power systems. However, this integration of numerous heterogeneous technologies and devices increases the attack surface. Therefore, a myriad of security techniques have been introduced based on technologies such as public key cryptosystems, blockchain, bilinear pairing and elliptic curve cryptography. However, majority of these protocols have security challenges while the others incur high complexities. Therefore, they are not ideal for some of the smart grid components such as smart meters which are resource-constrained. In this paper, a protocol that leverages on digital certificates, signatures, elliptic curve cryptography and blockchain is developed. The formal verification using Real-Or-Random (ROR) model shows that the derived session keys are secure. In addition, semantic security analysis shows that it is robust against typical smart grid attacks such as replays, forgery, privileged insider, side-channeling and impersonations. Moreover, the performance evaluation shows that our protocol achieves a 17.19% reduction in the computation complexity and a 46.15% improvement in the supported security and privacy features.

## 1. Introduction

The traditional power grid network faces numerous challenges regarding flexibility, energy utilization efficiency, safety and environmental protection [1]. This has led to the development of Smart Grids (SGs) which have advanced computing and sensing abilities using a number of sensors and actuators that generate and transmit real-time power related information in a bidirectional manner. In SGs, Information and communication Technologies (ICTs) are deployed to facilitate data exchange between Utility Service Providers (USPs) and the clients. This helps in the control, adjustments and optimization of power consumption based on real-time client needs as reported by the smart meters [1]. As explained in [2], SGs offer seamless integration of ICTs, distribution domains, cyber physical systems as well as power generation domains. Therefore, a typical smart grid comprises of automation technologies, power generation, distribution, transmission as well as advanced sensing and control components. These technologies help boost efficiency, reliability, transparency and flexibility of the electric power systems [3,4]. In addition, the advanced metering infrastructure, self-healing and demand response of the SGs result in optimum utilization of power stations as well as better control of consumer costs. The Smart Meter (SM) is the main component in the SGs and can generate real-time power consumption reports which are then periodically transmitted to the USPs. This normally happens after every 15 minutes. The analysis of these reports at the USPs facilitates the prediction of power demands as well as the adjustments in its generation and distribution. In so doing, the SGs reduce costs and energy consumptions while facilitating the integration of renewable energy sources [5].

Although the smart grid brings forth numerous merits in the face of increasing demand for electricity, these systems are vulnerable to numerous attacks. This situation is worsened by the many connected devices in a typical smart grid. Therefore, data confidentiality, integrity and authentication challenges are common in SGs. Authors in [6] attribute this to the heterogeneous connectivity in smart grid networks in which numerous Internet of Things (IoT) devices are incorporated to generate, distribute and transmit data in various systems such as smart meters and Supervisory Control And Data Acquisition (SCADA). In addition, the integration of ICTs in power systems has been noted in [7] to render the grid vulnerable to attacks such as impersonation, replay and Man-in-the Middle (MitM). As explained in [2], Demand Response Management (DRM) is crucial for improved reliability and efficiency smart grid ecosystem. This is normally enabled by the frequent data transfer between the USPs and smart meters. Unfortunately, these data transfers are prone to many threats such as tampering. This is made worse by the transmission of the data over insecure public channels [8,9]. Therefore, adversaries can intercept the communication process and recover consumer’s secret information. Consequently, the balancing of security, privacy, functionality and efficiency is one of the greatest challenges facing the SGs [10]. Authors in [6] explain that if data and device security are not handled properly, they can lead to grid failure.

In addition to security, user privacy leakage is another serious issue that must be solved in SGs. In this context, the adversaries can intercept electricity consumption data and try to associate it with particular users [11]. For instance, the tracking of power consumption patterns by various appliances may help attackers monitor consumer behavior, hobbies, future plans, and lifestyle as well as establish the status of home. This helps the attackers determine when to break-in and commit crimes [12]. It is evident that the large number of heterogeneous devices in the SGs exposes them to a myriad of security and privacy risks [13,14]. To counter these challenges, robust authentication must be executed to ensure that only authorized entities get access to system resources [15]. In addition, session keys must be established to facilitate secure message exchanges among the authenticated entities and uphold their privacy. Unfortunately, majority of the conventional authentication protocols are computationally intensive and hence not suitable for resource-limited smart grid networks [5].

### 1.1 Contributions

The major contributions of this paper included the following:

- We deploy digital signatures to preserve data integrity by preventing malicious tampering of the transmitted data. Since these signatures are verified at the receiver terminals, forgery and repudiation are thwarted.
- To preserve user privacy, the real identities of the users are never sent over the public channels. In addition, all exchanged messages are enciphered using the negotiated session keys to prevent attackers from eavesdropping the communication channel and obtain user sensitive information such as real-time power consumption reports.
- During transactions management, we validate all blocks before their addition to the blockchain. This makes it difficult for attackers to modify or corrupt the smart grid transactions.
- The performance evaluation is executed to show that the proposed scheme has the least computation complexity and relatively low communication costs. As such, our protocol is able to offer user privacy and real-time power consumption reports protection at improved efficiencies.
- Extensive security analysis is carried out to show that our scheme is provably secure. In addition, it is shown to support mutual authentication, key agreement, key secrecy, anonymity and untraceability. Moreover, it is demonstrated to be robust against typical smart grid attacks such as ephemeral secret leakage, eavesdropping, key escrow, session hijacking, KSSTI, replays, forgery, MitM, privileged insider, physical, side-channeling and impersonations.

The rest of this paper is structured as follows: Section 2 discusses the related works while Section 3 describes the proposed protocol. On the other hand, Section 4 presents the security analysis of the proposed protocol while Section 5 discusses its performance evaluation. Towards the end of this paper, Section 6 describes the conclusions and future works.

### 1.2 Motivation

The reliance on public channels for data exchanges in smart grids exposes these networks to numerous attacks such as replay, impersonation, forgery and MitM. In addition, the incorporation of ICTs has been shown to introduce numerous security threats to the SGs which can be exploited by adversaries. This might lead to the compromise of terminals such as smart meters which can then transmit falsified information to the grid, resulting in misleading data analytics, forecasting models and adjustments related to DRM. In addition, normal operations of the grid can be interfered with, or wrong power grid operations status can be fed to user terminals. Any successful interruptions on the access from smart meters to the metering system can render the control center unable to obtain real-time consumer load status, leading to power supply interruptions and grid collapse. It is also possible for attackers to monitor consumer load and correlate the time dimensions of diverse household appliances. This results in the determination of user behavioral patterns, personal preferences, activities and preferences, thereby infringing on personal privacy. Although many protocols have been developed to tackle these challenges, many of them are either vulnerable to security and privacy attacks or incur high computation [16] and communication overheads. Due to the hardware, storage capacity and computing power limitations of the smart grid components, they cannot execute highly complex cryptographic operations such as bilinear pairings. There is therefore need to develop an efficient protocol that will help address some of these performance and security issues.

### 1.3 Adversarial model

We deploy the widely accepted Canetti–Krawczyk (CK) threat model, in which an adversary is thought to have a range of capabilities that can compromise the smart grid communication process. The assumption in this model is that insecure public communication channels are utilized for message exchanges, and the Registration Authority (RA) is sufficiently protected. Therefore, adversary *Å* can eavesdrop the channel, intercept, alter, replay and delete the transmitted data but cannot compromise RA. In addition, *Å* can physically capture the smart grid components such as smart meters and use power analysis attacks to retrieve memory resident secrets. Moreover, session states and keys can be accessed by *Å*.

### 1.4 Key design principles

Smart grid faces numerous security, performance and privacy challenges that must be addressed. Therefore, many protocols have been developed over the recent past. For instance, to preserve privacy and integrity, aggregate signature based schemes have been presented. However, signature verification in these schemes incurs high computation complexities [11]. As explained in [17], majority of the current protocols fail to support flexible key management and conditional anonymity. In addition, most of the current authentication algorithms utilize the Rivest Shamir and Adleman (RSA) for asymmetric encryption of the digital signatures.

- Due to perfections and developments of large integer factorization, the required RSA algorithm key length has increased. Therefore, the encryption and decryption speeds have been reducing, making its hardware implementation difficult [14]. Fortunately, Elliptic Curve Encryption (ECC) algorithm attains the same enciphering strength as RSA but at shorter key lengths. Therefore, it can solve the challenges in RSA algorithm. ECC security is basically hinged on the problem of the Elliptic Curve Discrete Logarithm (ECDL) over the Galois fields. Mathematically, there is no sub-exponential algorithm to the ECDL problem. Since the chips in most of the smart grid devices have limited RAM size and processing power, the digital signatures must be implemented using public key cryptography algorithm with low computation overheads but strong encryption. As explained in [14], a 160-bit ECC algorithm offers the same level of security as the 1024-bit RSA algorithm, while a 210-bit ECC algorithm’s security level is equivalent to a 2048-bit RSA algorithm. Therefore, we adopt ECC in the proposed protocol.
- To protect the smart grid terminals, their identities and communication channels security are taken into consideration. We authenticate all terminals using digital certificates to uphold their legitimacy. On the other hand, confidentiality and integrity of the transmitted data in appendix A is protected via the negotiated session keys that are used to encipher the communication channel.
- The smart meters collect real-time data and upload it to the USPs to facilitate DRM, which is critical for the maintenance of smart grid demand and supply stability. Therefore, assigning the USPs an additional responsibility of transactions management increases their data processing pressure, communication load and system response latencies. Therefore, we reduce pressure at the USPs by incorporating the cloud servers and blockchain centers to management the smart meter transactions. This is due to their distributed nature, high storage capacity, computing power and low latencies.

### 1.5 Security and performance requirements

**Mutual authentication:** All the network entities must validate their identities before sharing their data.

**Session key agreement:** Upon successful mutual authentication, the communicating parties should negotiate session keys to encipher the exchanged data.

**Key secrecy:** An adversary in possession of the current session key should be unable to derive the keys for the previous as well as subsequent communication session.

**Anonymity and untraceability:** Attackers should be unable to discern the real identities of the smart grid entities upon eavesdropping the channel. In addition, it should be difficult to associate the captured data to any smart grid device or user.

**Formal verification:** The derived session keys for data enciphering should be mathematically secure.

**Attacks resilience:** To offer enhanced security and privacy protection, the proposed protocol should thwart conventional smart grid attacks such as ephemeral secret leakage, eavesdropping, key escrow, session hijacking, KSSTI, replays, forgery, MitM, privileged insider, physical, side-channeling and impersonations.

**Low complexities:** The smart grid supports high number of smart meters whose real-time power consumption data must be processed and responded to. Therefore, the proposed protocol must be lightweight to facilitate efficient processing of the massive smart meter data. This will ensure low network and processing latencies for delay-sensitive smart grid applications.

## 2. Related work

Efficient, reliable and secure communication procedures are crucial for the smart grid networks [18]. Therefore, many schemes have been put forward over the recent past. For instance, certificate based authentication protocols are presented in [2,19]. In addition, a certificate-based data aggregation technique is introduced in [20]. However, the demand response management scheme in [2] has high computation costs due to numerous elliptic curve point multiplications and has not been analyzed against attacks such as session hijacking, privileged insider and ephemeral secret leakage. Similarly, the security mechanisms in [19,20] have not been evaluated against attacks such as privileged insider and side-channeling. On its part, the scheme in [21] does not support untraceability and protection against attacks such as side-channeling. To offer enhanced security, blockchain-based schemes are developed in [5,22–26]. However, security analyses in [5,24] fail to include attacks such as privileged insider and ephemeral secret leakage. Similarly, security analysis of the scheme in [25] is missing while the privacy preserving technique in [22] is not evaluated against side-channeling and forgery attacks. On the other hand, the protocol in [23] is never analyzed against many attacks such as forgery while the scheme in [26] lacks formal security evaluation.

To support user privacy and data integrity, conventional blind signature based schemes have been developed in [27–29] while ring signature based protocol is introduced in [11]. Similarly, identity-based blind signature protocols are presented in [30–33] while signature and encryption technique is developed in [34]. In addition, group signature-based scheme is introduced in [35] while the protocol in [36] combines blind and group signatures to offer privacy protection. Moreover, certificate-less blind signature technique is developed in [1] while a certificate-based blind signature mechanism is presented in [37]. Although these signature-based schemes solve user data integrity issues, they are susceptible to quantum attacks [33]. In addition, most of these signature schemes have numerous security issues and some of them are inefficient due to bilinear pairing operations [1,38]. For instance, the scheme in [11] cannot offer key secrecy, untraceability and lacks formal verification. As explained in [11], group signature is facilitated by group administrator and hence may trace the identity of the group members. On its part, the scheme in [33] lacks semantic security analysis while the protocol in [1] has not been evaluated against attacks such as privileged insider,side-channeling and MitM. On the other hand, the protocol in [32] fails to offer support for trust measurement. Due to its requirement for the maintenance of the certificate revocation list, this approach incurs extra overheads. Although the scheme in [39] can address this problem, it relies on a third party for secure session establishment between smart grid devices.

To protect against various insider and outside attacks, an ECC-based scheme is developed in [40]. However, this scheme incurs extensive communication and computation overheads. To preserve privacy during data sharing, secure aggregation techniques are presented in [41–43]. Although the scheme in [41] does not depend on trusted third parties and can prevent collusion attacks, its fault tolerance is low [10] and the computation costs [44] at the smart meter side is high [3]. Similarly, the protocol in [42] can prevent collusion attacks but at high communication overheads and complicated key management procedures [10]. Although the Chebyshev chaotic maps based scheme in [45] addresses this problem, it lacks evaluation against forgery and session hijacking attacks. On its part, the protocol in [43] offers privacy protection and flexible user management at high computation costs due to frequent key updates for each time slot [3]. To preserve anonymity during the authentication process, bilinear pairing based security techniques are introduced [46,47]. Although the technique in [46] thwarts smart meter private key leakages, it only achieves one-way authentication which might expose intelligent terminals to malicious control and operation by adversaries. On its part, the protocol in [47] is susceptible to impersonation and ephemeral secret leakage attacks [48]. Due to the pairing operations, these two protocols have high computation costs [49]. This problem can be solved by the lightweight scheme in [50]. However, its versatility and communication complexity are increased due to the requirement that the USPs assign random nonces to the smart meters prior to each data collection. Although the protocol in [51] potentially solves this inefficiency issue, its formal security verification has not been done. To prevent cloning attacks, a physically unclonable function (PUF) based protocol are presented in [52–54]. However, PUF-based schemes have stability challenges. On the other hand, the schemes in [55,56] incur extensive computation overheads due to bilinear and scalar multiplications, respectively. Although the protocol in [57] is relatively lightweight, it is not evaluated against threats such as session hijacking and ephemeral secret leakage. Table 1 presents a summary of these current security solutions.

**Table 1:** Summary of related works.

Scheme	Technique	Gap (s)
[2]	Certificate	High computation costs
[5]	Blockchain	Not evaluated against such as privileged insider and ephemeral secret leakage
[21]	Signature	Fails to support untraceability and protection against attacks such as side-channeling
[19,20]	Certificate	Lack evaluation against attacks such as privileged insider & side-channeling
[22]	Blockchain	Not analyzed against side-channeling and forgery attacks
[23]	Blockchain	Lacks evaluation against forgery attacks
[24]	Blockchain	Not evaluated against such as privileged insider and ephemeral secret leakage
[25]	Blockchain	Lacks security analysis
[26]	Blockchain	Lacks formal security analysis
[11,27–37]	Signature	Susceptible to quantum attacks
[39]	ECC	Reliance on a third party for secure session establishment
[40]	ECC	High communication and computation overheads
[41]	Homomorphic	Low fault tolerance
[42]	Homomorphic	High communication overheads
[43]	Homomorphic	High computation costs
[46]	Bilinear pairing	Vulnerable to malicious control and operation
[47]	Bilinear pairing	Susceptible to impersonation & ephemeral secret leakage attacks
[50]	Hashing, XOR	High communication complexity
[51]	ECC	Formal security verification is not done
[52–54]	PUF	Stability challenges
[55]	Bilinear pairing	High computation costs
[56]	ECC	Extensive computation overheads
[57]	ECC	Not evaluated against threats such as ephemeral secret leakage and session hijacking.

It is evident that many protocols have been developed for security enhancement in smart grids. However, a number of security, performance and privacy issues still lurk in these schemes. The proposed protocol is shown to be efficient, privacy preserving and thwarts most of the attacks inherent in the above schemes.

## 3. The proposed scheme

**Fig 1:**
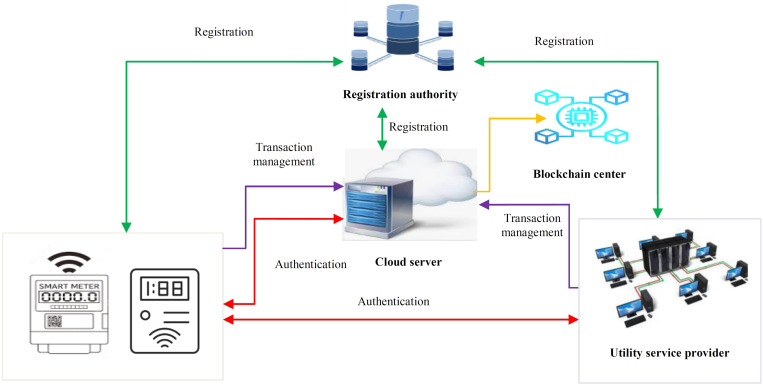
Network model.

The Smart Meter (SM), Service Provider (SP), the Registration Authority (RA) and the Cloud Servers (CSs) are the main components of the proposed protocol as shown in Fig 1. Here, the smart meter collects and forwards power consumption reports to the utility service provider.

However, all the smart meters and service provider must first register at the registration authority so that they are assigned the security tokens to use in the later phases. As already explained, we deploy cloud servers to offload the transaction management tasks from the service providers. Table 2 presents the symbols used throughut this paper.

**Table 2:** Symbols.

Symbol	Description
*G*	Base point in an elliptic curve
*ID*_RA_	Unique identity of the registration authority
*MK*_RA_	RA’s private master key
*PK*_RA_	RA’s public key
*E*_sig_	ECDSA signature generation algorithm
*E*_ver_	ECDSA signature verification algorithm
*D*_k_	Decryption using key *k*
*ID*_CS_	Unique identity of the cloud server
*PID*_CS_	Cloud server pseudo-identity
*T*_i_	Timestamp *i*
*∆T*	Maximum permissible transmission delay
*η*_i_	Random secret key *i*
ℂ_CS_	Cloud server certificate
*SK*_CS_	Cloud server secret key
*PK*_CS_	Cloud server public key
*ID*_SM_	Smart meter unique identity
*SK*_SM_	Smart meter secret key
*TID*_SM_	Transient smart meter identity
*ID*_SP_	Unique identity of the utility service provider
*TID*_SP_	Transient utility service provider identity
*SK*_SP_	Utility service provider secret key
||	Concatenation operation
ℝ_i_	Random nonce *i*
*ɸ*_SM,_ *ɸ*_SP,_ *ɸ*_SC_	Session keys
*h* (.)	Collision-resistant one-way hash function
^⊕^	XOR operation

Basically, our protocol comprises of 5 major phases, which include system setup, registration, mutual authentication and key negotiation, key and transactions management. The sub-sections below describe these phases in greater details.

### 3.1 System setup

The goal of this phase is to have the RA generate security parameters for all the network entities. These parameters are then deployed in the proceeding phases of the proposed protocol. For signature generation and verification, we deply the Elliptic Curve Digital Signature Algorithm (ECDSA). However, the Practical Byzantine Fault Tolerance (PBFT) is utilized as a consensus algorithm. Here, non-singular ellipic curve (NS-EC) and Galois field (GF) are utilized as described in the following steps.

**Step 1:** The RA generates *ID*_RA_ as its unique identity before selecting some large prime number *q* and NS-EC over the GF(*q*). Considering some two constants *a* and *b*, where *a*, *b*$\in Z_{q}=\{0,1,2,\ldots q-1\}$, then the condition 4*a*^3^ + 27*b*^2^ ≠  0 (mod *q*) must be satisfied. Here, NS-EC is of the form *E*_q_ (*a*,*b*): *y*^2^ =  *x*^3^ +  *ax* +  *b* (mod q).

**Step 2:** The RA picks *G* $\in E_{q}(a,b$as the base point, whose order is *g*, which is as large as *q*. Next, it chooses *h*(.) as some collision-resistant one way hashin*g* function. It also chooses *E*_sig_ and *E*_ver_ as ECDSA signature generation and verification algorithms respectively. Moreover, PBFT is chosen as the consensus algorithm.

**Fig 2:**
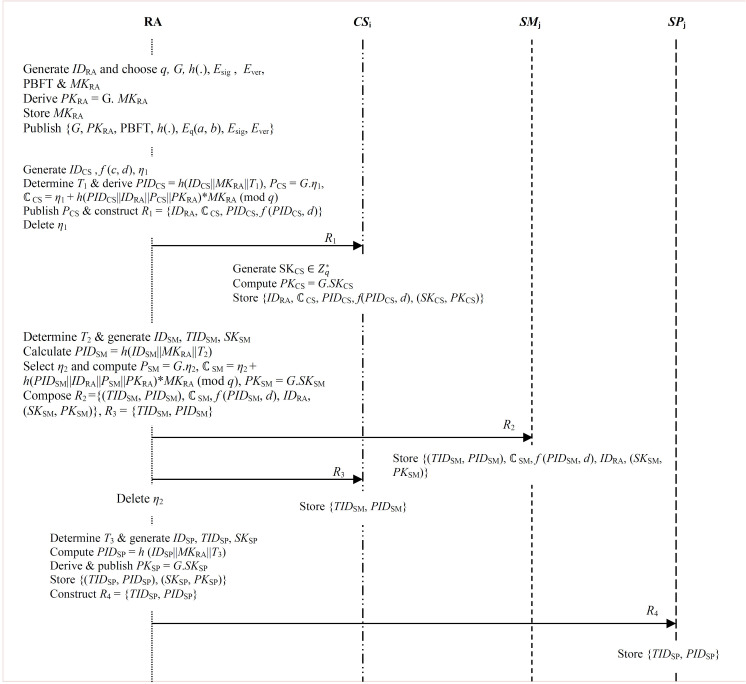
System setup and registration.

**Step 3:** The RA selects *MK*_RA_ $\in Z_{q}^{*}$as its secret master key before using this key to derive its corresponding public key *PK*_RA_ =  G. *MK*_RA_. At the end, the RA secretly stores *MK*_RA_ before publishing parameter set {*G*, *PK*_RA_, PBFT, *h*(.), *E*_q_(*a*, *b*), *E*_sig_, *E*_ver_} as shown in Fig 2.

### 3.2 Registration phase

The aim of this phase is to register the cloud server, smart meters and the utility service providers. This registration is carried out with the help of the RA and is one-time process which is described in the sub-sections below.

#### 3.2.1 Cloud server registration.

The following 3 steps are executed to register cloud server *CS*_i_ to the RA.

**Step 1:** The RA generates *CS*_i_ unique identity *ID*_CS_ and some symmetric *n*-degree bivariate polynomial *f* (*c*, *d*) over finite field GF(*q*). Here, $p(c,d=\sum_{i=0}^{n}\sum_{j=0}^{n}e_{ij}c^{i}d^{j},$ where *p* (*c*, *d*) =  *p* (*d*, *c*) and$e_{ij}\in Z_{q}$. The RA determines the current timestamp *T*_1_ and derives *CS*_i_ pseudo-identity *PID*_CS_ =  *h*(*ID*_CS_||*MK*_RA_||*T*_1_).

**Step 2:** The RA generates random secret key *η*_1_$\in Z_{q}^{*}$ that is used to compute its corresponding public key *P*_CS_ =  *G*.*η*_1_. Next, RA creates *CS*_i_ certificate as ℂ_CS_ =  *η*_1_ +  *h*(*PID*_CS_||*ID*_RA_||*P*_CS_||*PK*_RA_) * *MK*_RA_ (mod *q*). It then publishes *P*_CS_ before constructing registration message *R*_1_ =  {*ID*_RA_, ℂ_CS_, *PID*_CS_, *f* (*PID*_CS_, *d*)} that is forwarded to *CS*_i_ over secure channels. Finally, the RA deletes random secret key *η*_1_ as shown in Fig 2.

**Step 3:** Upon receiving registration message *R*_1_ from RA, *CS*_i_ proceeds to generate its secret key

*SK*_CS_ $\in Z_{q}^{*}$ and computes its corresponding public key *PK*_CS_ =  *G*.*SK*_CS_. Finally, *CS*_i_ stores parameter set {*ID*_RA_, ℂ_CS_, *PID*_CS_, *f*(*PID*_CS_, *d*), (*SK*_CS_, *PK*_CS_)}.

#### 3.2.2 Smart meter registration.

The following 4 steps are carried out during the registration of smart meter *SM*_j_ to the RA.

**Step 1:** The RA determines the current timestamp *T*_2_ and generates smart meter unique identity *ID*_SM_ that is used to derive its pseudo-identity *PID*_SM_ =  *h*(*ID*_SM_||*MK*_RA_||*T*_2_). Next, it generates its random transient identity *TID*_SM_.

**Step 2:** RA chooses some random private key *η*_2_ $\in Z_{q}^{*}$ and derives its corresponding public key *P*_SM_ =  *G*.*η*_2_. This is followed by the generation of *SM*_j_ certificate ℂ_SM_ =  *η*_2_ +  *h*(*PID*_SM_||*ID*_RA_||*P*_SM_||*PK*_RA_) * *MK*_RA_ (mod *q*).

**Step 3:** The RA generates secret key *SK*_SM_$\in Z_{q}^{*}$ together with its corresponding public key *PK*_SM_ =  *G*.*SK*_SM_ Next, it composes registration message *R*_2_ = {(*TID*_SM_, *PID*_SM_), ℂ_SM_, *f* (*PID*_SM_, *d*), *ID*_RA_, (*SK*_SM_, *PK*_SM_)} that is forwarded to the *SM*_j_ for safe storage of this parameter set as shown in Fig 2.

**Step 4:** RA composes registration message *R*_3_ =  {*TID*_SM_, *PID*_SM_} that is forwarded to *CS*_i_ over secure channels. Finally, the RA erases random secret key *η*_2_.

#### 3.2.3 Utility service provider.

The following 3 steps are involved during the registration of utility service provider *SP*_j_.

**Step 1:** The RA determines current timestamp *T*_3_ and selects some unique identity *ID*_SP_ for *SP*_j_ that is used to compute its pseudo-identity *PID*_SP_ =  *h* (*ID*_SP_||*MK*_RA_||*T*_3_). Next, it chooses random transient identity *TID*_SP_ for *SP*_j_.

**Step 2:** The RA generates secret key *SK*_SP_$\in Z_{q}^{*}$ and equivalent public key *PK*_SP_ =  *G*.*SK*_SP_. This is followed by the publishing of *PK*_SP_.

**Step 3:** RA securely stores parameter set {(*TID*_SP_, *PID*_SP_), (*SK*_SP_, *PK*_SP_)}. Next, it constructs registration message *R*_4_ =  {*TID*_SP_, *PID*_SP_} that is sent to the associatated *SP*_j_ over secure channels as shown in Fig 2.

### 3.3 Mutual authentication and key negotiation

**Fig 3:**
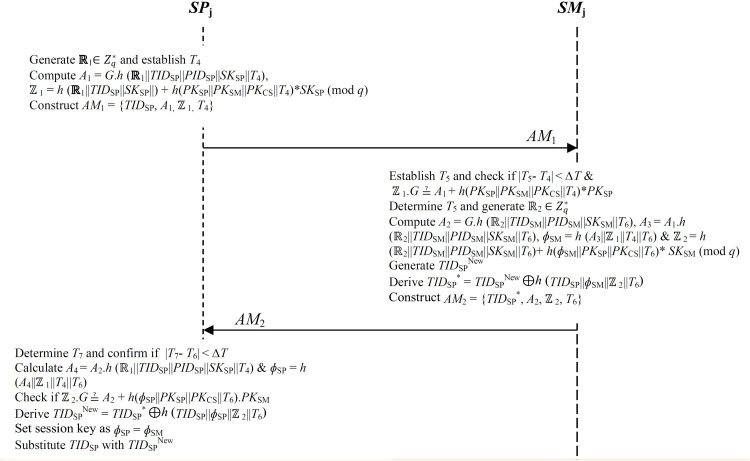
Mutual authentication and key negotiation.

During the mutual authentication between *SP*_j_ and *SM*_j_, steps 1–6 are executed. Fig 3 presents a summary of the message exchanges during these procedures.

**Step 1:** The *SP*_j_ generates random nonce **ℝ**_1_$\in Z_{q}^{*}$ and determines the current timestamp *T*_4_. Next, it derives *A*_1_ =  *G*.*h* (**ℝ**_1_||*TID*_SP_||*PID*_SP_||*SK*_SP_||*T*_4_) as well as signature ℤ_1_ =  *h* (**ℝ**_1_||*TID*_SP_||*SK*_SP_||) +  *h*(*PK*_SP_||*PK*_SM_||*PK*_CS_||*T*_4_) * *SK*_SP_ (mod *q*). Finally, it composes message authenticatiom message *AM*_1_ =  {*TID*_SP_, *A*_1,_ ℤ_1,_ *T*_4_} that is sent over to *SM*_j_ over public channels as shown in Fig 3.

**Step 2:** Upon receiving authentication message *AM*_1_, *SM*_j_ determines current timestamp *T*_5_ and checks if | *T*_5_- *T*_4_ | < *∆ T*. Basically, the session is aborted when this verification fails. Otherwise, *SM*_j_ validates signature ℤ_1_ by confirming whether ℤ_1_.*G* ≟  *A*_1_ +  *h*(*PK*_SP_||*PK*_SM_||*PK*_CS_||*T*_4_) * *PK*_SP_. Provided that this confirmation is valid, *SM*_j_ determines the current timestamp *T*_6_ and generates random nonce ℝ_2_$\in Z_{q}^{*}$.

**Step 3:** The *SM*_j_ derives *A*_2_ =  *G.h* (ℝ_2_||*TID*_SM_||*PID*_SM_||*SK*_SM_||*T*_6_) and *A*_3_ =  *A*_1_.*h* (ℝ_2_||*TID*_SM_||*PID*_SM_||*SK*_SM_||*T*_6_) as well as session key *ɸ*_SM_ =  *h* (*A*_3_||ℤ_1_||*T*_4_||*T*_6_). Next, it computes the signature on ℝ_2_ and *ɸ*_SM_ as ℤ_2_ =  *h* (ℝ_2_||*TID*_SM_||*PID*_SM_||*SK*_SM_||*T*_6_) + *h*(*ɸ*_SM_||*PK*_SP_||*PK*_CS_||*T*_6_) * *SK*_SM_ (mod *q*).

**Step 4:** *SM*_j_ generates a new transient identity *TID*_SP_^New^ for *SP*_j_. Next, it computes *TID*_SP_^ *^ =  *TID*_SP_^New ⊕ *h* (^*TID*_SP_||*ɸ*_SM_||ℤ_2_||*T*_6_). Finally, it composes authentication message *AM*_2_ =  {*TID*_SP_^ * ^, *A*_2_, ℤ_2_, *T*_6_} that is transmitted over to the *SP*_j_ through public channels as shown in Fig 3.

**Step 5:** On receiving authentication message *AM*_2_ at timestamp *T*_7_, the *SP*_j_ checks if | *T*_7_- *T*_6_ | < *∆ T*. Provided that this verification fails, the session is terminated. Otherwise, it computes *A*_4_ =  *A*_2_.*h* (ℝ_1_||*TID*_SP_||*PID*_SP_||*SK*_SP_||*T*_4_) and session key *ɸ*_SP_ =  *h* (*A*_4_||ℤ_1_||*T*_4_||*T*_6_).

**Step 6:** The *SP*_j_ validates signature ℤ_2_ by confirming if ℤ_2_.*G* ≟  *A*_2_ +  *h*(*ɸ*_SP_||*PK*_SP_||*PK*_CS_||*T*_6_). *PK*_SM_. Provided that this validation succeeds, the *SP*_j_ computes *TID*_SP_^New^ =  *TID*_SP_^ * ⊕ *h* (^*TID*_SP_||*ɸ*_SP_||ℤ_2_||*T*_6_^). Finally, it substitutes^ *TID*_SP_ with its updated version *TID*_SP_^New^ in its repository.

### 3.4 Key management

The goal of this phase is to secure the transactions transmitted to the cloud servers from any of the smart grid device. For a given utility service area *U*_SA_ (*SA = 1,2,3,….N*), steps 1–6 are carried out to setup the session keys between the cloud server *CS*_i_ and any smart device such as *SM*_j_.

**Fig 4:**
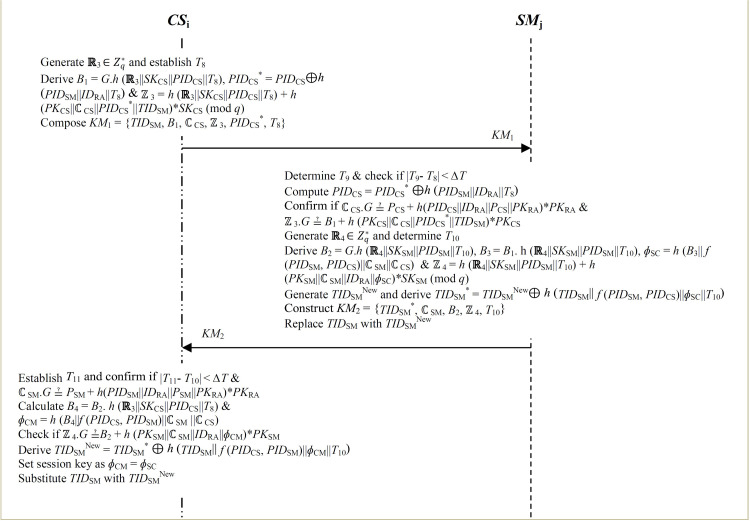
Key management.

**Step 1:** The *CS*_i_ generates random nonce **ℝ**_3_ $\in Z_{q}^{*}$ and determines the current timestamp *T*_8_. Next, it derives *B*_1_ =  *G*.*h* (**ℝ**_3_||*SK*_CS_||*PID*_CS_||*T*_8_), *PID*_CS_^ *^ =  *PID*_CS_^ ⊕ *h* (^*PID*_SM_||*ID*_RA_||*T*_8_^) and^ ℤ_3_ =  *h* (**ℝ**_3_||*SK*_CS_||*PID*_CS_||*T*_8_) +  *h* (*PK*_CS_||ℂ_CS_||*PID*_CS_^*^||*TID*_SM_) * *SK*_CS_ (mod *q*). Finally, it constructs key management message *KM*_1_ =  {*TID*_SM_, *B*_1_, ℂ_CS_, ℤ_3_, *PID*_CS_^ * ^, *T*_8_} that is forwarded towards *SM*_j_ over public channels as shown in Fig 4.

**Step 2:** Upon receiving message *KM*_1_ at timestamp *T*_9_, *SM*_j_ confirms whether | *T*_9_- *T*_8_ | < *∆ T*. On condition that this verification is successful, *SM*_j_ derives *PID*_CS_ =  *PID*_CS_^ * ⊕ *h* (^*PID*_SM_||*ID*_RA_||*T*_8_). Next, it validates the received certificate ℂ_CS_, signature ℤ_3_ and *TID*_SM_ by checking if ℂ_CS_.*G* ≟  *P*_CS_ +  *h*(*PID*_CS_||*ID*_RA_||*P*_CS_||*PK*_RA_) * *PK*_RA_ and ℤ_3_.*G* ≟  *B*_1_ +  *h* (*PK*_CS_||ℂ_CS_||*PID*_CS_^*^||*TID*_SM_) * *PK*_CS_. Provided that these conditions do not hold, the session is aborted. Otherwise, *SM*_j_ generates random nonce **ℝ**_4_ $\in Z_{q}^{*}$and determines the current timestamp *T*_10_.

**Step 3:** The *SM*_j_ derives *B*_2_ =  *G*.*h* (**ℝ**_4_||*SK*_SM_||*PID*_SM_||*T*_10_), *B*_3_ =  *B*_1_. h (**ℝ**_4_||*SK*_SM_||*PID*_SM_||*T*_10_), *ɸ*_SC_ =  *h* (*B*_3_|| *f* (*PID*_SM_, *PID*_CS_)||ℂ_SM_||ℂ_CS_) and ℤ_4_ =  *h* (**ℝ**_4_||*SK*_SM_||*PID*_SM_||*T*_10_) +  *h* (*PK*_SM_||ℂ_SM_||*ID*_RA_||*ɸ*_SC_) * *SK*_SM_ (mod *q*).

**Step 4:** The *SM*_j_ generates *TID*_SM_^New^ and computes *TID*_SM_^ *^ =  *TID*_SM_^New ⊕ *h* (^*TID*_SM_^||^ *f* (*PID*_SM_, *PID*_CS_)||*ɸ*_SC_||*T*_10_). It then composes key management message *KM*_2_ =  {*TID*_SM_^ * ^, ℂ_SM_, *B*_2_, ℤ_4_, *T*_10_} that is transmitted over to *CS*_i_ via public channels. Finally, *SM*_j_ substitutes *TID*_SM_ with its updated version *TID*_SM_^New^.

**Step 5:** On receiving message *KM*_2_ at timestamp *T*11, the *CS*_i_ checks if | *T*_11_- *T*_10_ | < *∆ T* such that the session is aborted upon validation failure. Otherwise, it validates the received certificate ℂ_SM_ by confirming whether ℂ_SM_.*G* ≟  *P*_SM_ +  *h*(*PID*_SM_||*ID*_RA_||*P*_SM_||*PK*_RA_) * *PK*_RA_. Provided that this verification is unsuccessful, the session is terminated. Otherwise, it derives *B*_4_ =  *B*_2_. *h* (**ℝ**_3_||*SK*_CS_||*PID*_CS_||*T*_8_) and session key *ɸ*_CM_ =  *h* (*B*_4_||*f* (*PID*_CS_, *PID*_SM_)||ℂ_SM_ ||ℂ_CS_).

**Step 6:** *CS*_i_ validates signature ℤ_4_ by checking if ℤ_4_.*G* ≟ *B*_2_ +  *h* (*PK*_SM_||ℂ_SM_||*ID*_RA_||*ɸ*_CM_) * *PK*_SM_. If this verification is successful, it derives *TID*_SM_^New^ =  *TID*_SM_^ * ⊕  *h* (^*TID*_SM_^||^ *f* (*PID*_CS_, *PID*_SM_)||*ɸ*_CM_||*T*_10_). Next, both the *CS*_i_ and *SM*_j_ sets their respective session key for payload enciphering and substitutes *TID*_SM_ with its update version *TID*_SM_^New^ in its repository.

### 3.5 Transactions management

**Fig 5:**
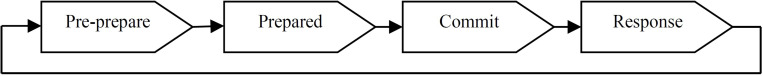
The PBFT consensus algorithm.

The data such as in appendix A collected by the smart devices in the smart grid system are regarded as being private and confidential. As such, the data from all the utility service provider coverage area are maintained in the private blockchain. In the proposed protocol, transactions are maintained in form of connected chain of blocks stored in the cloud servers. At each particular moment, the voting based consensus algorithm is deployed to ensure that each cloud server holds a similar copy of blockchain *BC*. Since most of the smart devices in the smart grid system are limited in terms of computation power, they cannot be charged with the creation of transactions for the blockchain. Therefore, the cloud servers are assigned this task since they have superior computational and storage resources. The four major phases in the PBFT consensus algorithm are depicted in Fig 5 below.

Using this consensus algorithm the four steps below describe the process of block addition and verification.

**Step 1:** The smart meters and cloud servers deploy session keys *ɸ*_CM_ and *ɸ*_SC_ already negotiated in Section 3.4 above to exchange all the collected data of appendix A. Thereafter, for a given block *β*_n_, *CS*_i_ makes *κ*_*τ*_ transactions (*Ψ*_1_, *Ψ*_2_, *Ψ*_3_, …, *Ψκ*_*τ*_). Next, *CS*_i_ enciphers these transactions using *PK*_CS_ as {$E_{{PK}_{CS}}\left(\Psi_{1}\right),E_{{PK}_{CS}}\left(\Psi_{2}\right),E_{{PK}_{CS}}\left(\Psi_{3}\right),\ldots .E_{{PK}_{CS}}\left(\Psi_{\kappa_{\tau }}\right\}$.

**Fig 6:**
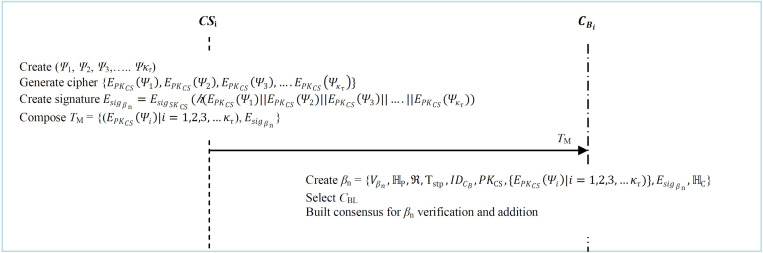
Transactions management.

**Step 2:** *CS*_i_ uses its secret key *SK*_CS_ to create a digital signature of the *κ*_*τ*_ transactions as $E_{{sig}_{\beta_{n}}}=E_{{sig}_{{SK}_{CS}}}(h(E_{{PK}_{CS}}(\Psi_{1})||E_{{PK}_{CS}}(\Psi_{2})||E_{{PK}_{CS}}\left(\Psi_{3}\right)||\ldots .||E_{{PK}_{CS}}(\Psi_{\kappa_{\tau }}))$. Next, it constructs transaction management message *T*_M_ =  {($E_{{PK}_{CS}}\left(\Psi_{i}\right)|i=1,2,3,\ldots \kappa_{\tau }$),$E_{{sig}_{\beta_{n}}}$} that basically uses to forward these enciphered *κ*_*τ*_ transactions to the blockchain center $C_{B_{i}}$ as shown in Fig 6.

**Fig 7:**
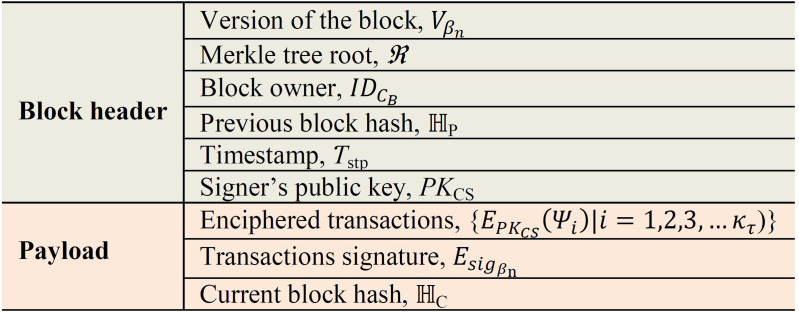
Structure of block *β*_n._

**Step 3:** Upon receiving message *T*_M_, the *C*_B_ creates block *β*_n_. Basically *β*_n_ contains details such as the previous block hash ℍ_P_, the current block hash ℍ_C_, signature $E_{{sig}_{\beta_{n}}}$, enciphered transactions *κ*_*τ*_, Merkle tree root *ℜ* on *κ*_*τ*_, as well the public key *PK*_CS_ for *CS*_i_. The detailed structure of *β*_n_ is depicted in Fig 7 below.

**Step 4:** Upon the formation of *β*_n_ at the $C_{B_{i}}$, the leader selection algorithm is invoked to choose the leader. Next, consensus is built for block verification and addition to the blockchain as detailed in Algorithm 1.

During consensus building, each of the blockchain centers $C_{B_{i}}$ is characterized by a pair of public-secret key pair {${SK}_{C_{B_{i}}},{PK}_{C_{B_{i}}}$}. Here, ${SK}_{C_{B_{i}}}\in Z_{q}^{*}$ is the secret key while ${PK}_{C_{B_{i}}}$ =  *G*.${SK}_{C_{B_{i}}}$ is its corresponding public key. Basically, ${PK}_{C_{B_{i}}}$ of all blockchain centers are known to each other. As shown in Algorithm 1, the inputs to the consensus process include number of faulty nodes *Χ*_F_ in the $C_{B_{i}}$, *β*_n_ and {${SK}_{C_{B_{i}}},{PK}_{C_{B_{i}}}$}. Here, the leader $C_{B_{i}}$ is denoted by *C*_BL_ and one of its responsibilities is to generate voting requests *V*_rs_. Therefore, it initially generates numeous enciphered voting requests *V*_RS_ utilizing the public keys of the receiver $C_{B_{i}}$, denoted as *C*_BR_. In addition, it maintains some valid vote counter *C*_L_ for the received votes. Thereafter, *C*_BL_ signs these *V*_RS_ before forwarding them to the respective followers *C*_BRs_ together with *β*_n_. Upon receiving the signed *V*_RS_, the *C*_BR_ verifies the signature ${\mathbb{Z}}_{V_{rs}}$in this request, deciphers it using its ${SK}_{C_{B_{i}}}$and validates the timestamp $T_{C_{BR}}$in *V*_RS_, *ℜ* as well as ℍ_P_.

Provided that these validations are successful, *C*_BR_ forwards its signature, voting response *V*_r_ along with the status of this verification *V*_S_ to the *C*_BL_. Here, *V*_r_ is encrypted using the public key${PK}_{C_{BL}}$ of *C*_BL_.

**Algorithm 1:** Consensus for *β*_n_ verification and addition.

After getting this response, *C*_BL_ validates the *C*_BR_’s signature before counting the votes maintained by *C*_L_. This happens only when both *V*_r_ is valid and *β*_n_ validation is successful. Upon receiving all the responses, *C*_BL_ checks whether $C_{L}\;1+2*X_{F}$. Provided that this condition holds, *C*_BL_ sends a commit block command *C*_BC_ to all *C*_BRs_. Consequently, *β*_n_ is appended to the distributed ledgers of all the peer nodes.

### 3.6 Secure addition of new smart grid devices

**Fig 8:**
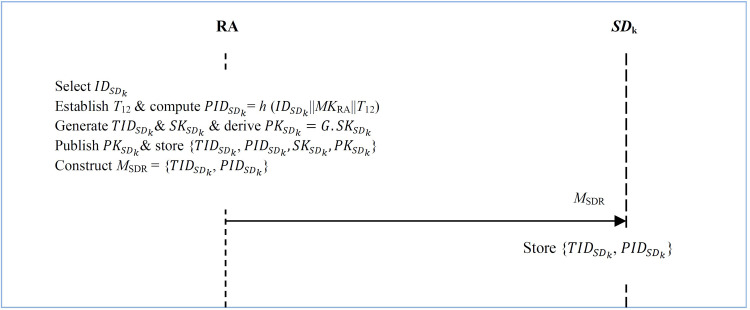
New smart grid devices addition.

In this phase, we detail how additional smart devices such as *SD*_k_ may be incorporated into the existing smart grid network. This is a 4-step process as described below and summarized in Fig 8.

**Step 1:** The RA chooses some unique real identity ${ID}_{{SD}_{k}}$ for *SD*_k_. Next, it determines current timestamp and *T*_12_ derives the pseudo-identity for *SD*_k_ as ${PID}_{{SD}_{k}}$ =  *h* (${ID}_{{SD}_{k}}$||*MK*_RA_||*T*_12_) as shown in Fig 8.

**Step 2:** RA generates random transient identity ${TID}_{{SD}_{k}}$. This is followed by the derivation of its secret key ${SK}_{{SD}_{k}}\in Z_{q}^{*}$. Next, it computes its corresponding public key as ${PK}_{{SD}_{k}}=G.{SK}_{{SD}_{k}}$.

**Step 3:** The RA makes ${PK}_{{SD}_{k}}$public before securely storing parameter set {${TID}_{{SD}_{k}}$,${PID}_{{SD}_{k}},{SK}_{{SD}_{k}},{PK}_{{SD}_{k}}$} in its repository.

**Step 4:** RA constructs smart device registration message *M*_SDR_ =  {${TID}_{{SD}_{k}}$,${PID}_{{SD}_{k}}$} that is forwarded to the smart device *SD*_k_.

## 4. Security analysis

In this section, the formal and informal security analysis of the proposed protocol ae presented. The sub-sections below describe these process in greater details.

### 4.1 Formal security analysis

In this section, we deploy the oracle model Real-Or-Random (ROR) to demonstrate that provable secure nature of the derived session keys. In our scheme, session keys are derived between utility service provider *SP*_j_ and smart meter *SM*_j_, as well as between cloud server *CS*_i_ and any smart device within the smart grid, such as smart meter *SM*_j_. We denote the adversary as *Å*, which is capable of launching *Execute* (), *Corrupt* (), *Reveal* () and *Test* () queries. Taking *O*_T_ as an arbitrary outcome of a flipped a fair coin *ε*, these queries are described in more detail in Table 3. In addition to these queries, *h* (.) is modeled as random oracle *Hash* which is available to *Å* as well as all other network entities *SM*_j_, *SP*_j_ and *CS*_i_. We denote the *i*^th^, *j*^th^ and *k*^th^ instances (random oracles) of *SM*_j_, *SP*_j_ and *CS*_i_ as $\Gamma_{SM}^{i},\Gamma_{SP}^{j}$ and $\Gamma_{CS}^{k}$. Suppose that instant $\Gamma^{m}$ receives the final legitimate exchanged message. In this case, $\Gamma^{m}$ is regarded as being in an accepted state. In addition, we denote the sequential ordering of all the exchanged messages in a given communication session as *Ṡ*. Basically, *Ṡ* becomes the session identifier of $\Gamma^{m}$ for this particular communication session. When random oracles $\Gamma^{p}$ and $\Gamma^{q}$ are mutual associates of each other, share the same *Ṡ* for mutual authentication and both are in accepted states, then they become associates to each other.

**Table 3:** Adversarial queries.

Query	Rationale
***Reveal* (**$\Gamma^{m}$)	Carried out by *Å* disclose session key *ɸ*_SP_ = *ɸ*_SM_ as well as *ɸ*_CM_ = *ɸ*_SC_ between $\Gamma^{m}$ and its respective associates
**Execute (**$\Gamma_{SM}^{i},\Gamma_{SP}^{j}$,$\Gamma_{CS}^{k}$)	Executed by *Å* to intercept messages exchanged among *SP*_j_, *SM*_j_ and *CS*_i_
**Test (**$\Gamma^{m}$)	Performed by *Å* using *O*_T_ to verify the disclosed session keys *ɸ*_SP_ = *ɸ*_SM_ as well as *ɸ*_CM_ = *ɸ*_SC_
**Corrupt (**$\Gamma_{SM}^{i},\Gamma_{SP}^{j}$)	Implemented by *Å* to extract secret tokens stored in compromised *SM*_j_ and *SP*_j_ respectively

Suppose that *SP*_j_ and *SM*_j_ share session key *ɸ*_SP_ =  *ɸ*_SM_ between them. Then, random oracle $\Gamma_{SM}^{i}$or $\Gamma_{SP}^{j}$ is regarded as being fresh this session key is unknown to *Å* even after executing the *Reveal* ($\Gamma^{m}$) query. Similarly, random oracle $\Gamma_{SM}^{i}$or $\Gamma_{CS}^{k}$ is fresh if session key *ɸ*_CM_ =  *ɸ*_SC_ remains unknown to *Å* even after executing the *Reveal* ($\Gamma^{m}$) query. We let *p*_t_ denote polynomial time and *λ*_CSB_ as the proposed certificate, signature and blockchain (CSB) based protocol. In this scenario, the advantage that *Å* (running in *p*_t_) has of breaking *λ*_CSB_’s semantic security is represented as ${Adv}_{\overset{˚}{A}}^{\lambda_{CSB}}(p_{t})$. This basically involves the compromise of session key *ɸ*_SP_ =  *ɸ*_SM_ established between *SM*_j_ and *SP*_j_, as well as *ɸ*_CM_ =  *ɸ*_SC_ negotiated between *SM*_j_ and *CS*_i_ during a given session. Taking *ε* and ε^*^ as valid and guessed bits respectively, then,


$$\begin{array}{c} {Adv}_{\overset{˚}{A}}^{\lambda_{\text{CSB}}}(p_{t})=|2Pr[\varepsilon *=\varepsilon ]-1| \end{array}$$ (1)

Suppose that the Elliptic Curve Decisional Diffie-Hellman Problem (ECDDHP), volume of *Hash* () queries and range space of *h* (.) are represented by *ω*, |*μ*| and *H*_n_ respectively. Using these notations, the advantage that adversary *Å* (running in *p*_t_) has in breaking *ω* is denoted as ${Adv}_{\overset{˚}{A}}^{\omega }(p_{t})$.

With the above notations, the following hypothesis can be stated.

**Hypothesis 1**: Suppose that *Å* is running in polynomial time *p*_t_ and wants to derive session key *ɸ*_SP_ =  *ɸ*_SM_ negotiated between *SP*_j_ and *SM*_j_, as well as session key *ɸ*_CM_ =  *ɸ*_SC_ established between *SM*_j_ and *CS*_i_ during a certain communication session. Therefore,


$$\begin{array}{c} {Adv}_{\overset{˚}{A}}^{\lambda_{\text{CSB}}}\left(p_{t}\right)\leq \frac{H_{n}^{2}}{\left|\mu \right|}+2({Adv}_{\overset{˚}{A}}^{\omega }(p_{t})) \end{array}$$ (2)

**Proof:** We deploy three games (denoted by *Ġm*_k_, where *k* =  0, 1, 2) to proof the above stated hypothesis. Suppose that ${Suc}_{{\dot{G}m}_{k}}$denotes an incident of *Å* winning *Ġm*_k_ via the guessing of valid bit *ε*. Therefore, the advantage or success probability of *Å* winning *Ġm*_k_ becomes


$$\begin{array}{c} {Adv}_{\overset{˚}{A},{\dot{G}m}_{k}}^{\lambda_{\text{CSB}}}\left(p_{t}\right)=Pr[{Suc}_{{\dot{G}}_{k}}] \end{array}$$ (3)

Thereafter, the following three adversarial games are played by *Å* in an effort to break the negotiated session keys.

***Ġm***_**0**_: In this game, adversary *Å* carries out the actual attack against the proposed protocol $\lambda_{CSB}$. Initially, *Å* chooses some random bit *ε*. Based on equation (1),

${Adv}_{\overset{˚}{A}}^{\lambda_{CSB}}\left(p_{t}\right)=|2({Adv}_{\overset{˚}{A},{\dot{G}m}_{0}}^{\lambda_{CSB}}\left(p_{t}\right)-1)$| (4)

***Ġm***_**1**_: The aim of this game is for *Å* to eavesdrop the communication channel. To accomplish this objective, *Å* carries out the *Execute* () query to intercept messages *AM*_1_ =  {*TID*_SP_, *A*_1,_ ℤ_1,_ *T*_4_}, *AM*_2_ =  {*TID*_SP_^ * ^, *A*_2_, ℤ_2_, *T*_6_}, *KM*_1_ =  {*TID*_SM_, *B*_1_, ℂ_CS_, ℤ_3_, *PID*_CS_^ * ^, *T*_8_} and ^*KM*2 =  {^*TID*_SM_^ * ^, ℂ_SM_, *B*_2_, ℤ_4_, *T*_10_}. Next, *Å* performs *Reveal* () and *Test* () queries with the aim of establishing whether the derived session keys are valid or just some stochastic parameters. However, the derivation of these keys requires a combination of long terms as well as short term security tokens. Due to the difficulties of compromising these tokens using the eavesdropped messages *AM*_1_, *AM*_2_, *KM*_1_ and ^*KM*2, the probability that^ *Å* has in successfully winning *Ġm*_1_ remain the same as that of *Ġm*_0_. Therefore,


$$\begin{array}{c} {Adv}_{\overset{˚}{A},{\dot{G}m}_{1}}^{\lambda_{\text{CSB}}}\left(p_{t}\right)={Adv}_{\overset{˚}{A},{\dot{G}m}_{0}}^{\lambda_{\text{CSB}}}\left(p_{t}\right) \end{array}$$ (5)

***Ġm***_**2**_: The ultimate goal of this game is to perform some active attack on *λ*_CSB_. To attain this goal, three queries and an attempt to solve *ω* are carried out. The executed queries include *Corrupt* (*SP*_j_), *Hash* () and *Corrupt* (*SM*_j_). The assumption made is that *Å* has already eavesdropped all the exchanged messages, including *AM*_1_, *AM*_2_, *KM*_1_ and ^*KM*2^. At the start, *Å* tries to compute *ɸ*_SM_ =  *h* (*A*_3_||ℤ_1_||*T*_4_||*T*_6_) and *ɸ*_SC_ =  *h* (*B*_3_|| *f* (*PID*_SM_, *PID*_CS_)||ℂ_SM_||ℂ_CS_). However, this requires that *Å* correctly derives *A*_1_ =  *G*.*h* (**ℝ**_1_||*TID*_SP_||*PID*_SP_||*SK*_SP_||*T*_4_), *A*_2_ =  *G.h* (ℝ_2_||*TID*_SM_||*PID*_SM_||*SK*_SM_||*T*_6_), *B*_1_ =  *G*.*h* (**ℝ**_3_||*SK*_CS_||*PID*_CS_||*T*_8_) and *B*_2_ =  *G*.*h* (**ℝ**_4_||*SK*_SM_||*PID*_SM_||*T*_10_) among other tokens. Evidently, each of these parameters by the collision-resistant one-way hashing function *h* (.) and hence attacker *Å* needs to solve *ω* in polynomial time *p*_t_. As already demonstrated, the success probability of *Å* in solving *ω* in polynomial time *p*_t_ is ${Adv}_{\overset{˚}{A}}^{\omega }(p_{t})$. It is also clear that these parameters also incorporate timestamps, short term secrets (such as random nonces) and long term secrets (such as private keys). To check for collisions in the message digests incorporated in the eavesdropped messages (*AM*_1_, *AM*_2_, *KM*_1_ and ^*KM*2^), adversary *Å* executes the *Hash* () query. However, since the chosen *h* (.) is collision-resistant, the success of this query is negligible. As such, the exclusion of these four queries renders *Ġm*_2_ and *Ġm*_1_ indistinguishable. To find the hash collision, the birthday paradox is applied, yielding the following:


$$\begin{array}{c} {Adv}_{\overset{˚}{A},{\dot{G}m}_{1}}^{\lambda_{\text{CSB}}}\left(p_{t}\right)-{Adv}_{\overset{˚}{A},{\dot{G}m}_{2}}^{\lambda_{\text{CSB}}}\left(p_{t}\right)\leq \frac{H_{n}^{2}}{2|\mu |}+{Adv}_{\overset{˚}{A}}^{\omega }(p_{t}) \end{array}$$ (6)

Upon adversarial execution of all these three games, *Å* finally attempts to guess the correct bit *ε* so as to win the game. Therefore,


$$\begin{array}{c} {Adv}_{\overset{˚}{A},{\dot{G}m}_{2}}^{\lambda_{\text{CSB}}}\left(p_{t}\right)=\frac{1}{2} \end{array}$$ (7)

Based on the semantic security definition of the proposed protocol in equation (4),

${\frac{1}{2}Adv}_{\overset{˚}{A}}^{\lambda_{CSB}}\left(p_{t}\right)=|({Adv}_{\overset{˚}{A},{\dot{G}m}_{0}}^{\lambda_{CSB}}\left(p_{t}\right)-\frac{1}{2})$| (8)

Using the triangular inequality, equations (5), (6) and (7) on equation (8) yields the following:


$$\begin{array}{c} \frac{1}{2}{Adv}_{\overset{˚}{A}}^{\lambda_{\text{CSB}}}\left(p_{t}\right)=\left|{Adv}_{\overset{˚}{A},{\dot{G}m}_{0}}^{\lambda_{\text{CSB}}}\left(p_{t}\right)-{Adv}_{\overset{˚}{A},{\dot{G}m}_{2}}^{\lambda_{\text{CSB}}}\left(p_{t}\right)\right| \end{array}$$

$$=\left|{Adv}_{\overset{˚}{A},{\dot{G}m}_{1}}^{\lambda_{CSB}}\left(p_{t}\right)-{Adv}_{\overset{˚}{A},{\dot{G}m}_{2}}^{\lambda_{CSB}}\left(p_{t}\right)\right|$$

$$\leq \frac{H_{n}^{2}}{2|\mu |}+{Adv}_{\overset{˚}{A}}^{\omega }(p_{t})$$ (9)

Multiplying the left hand side (LHS) and right hand side (RHS) by 2 yields the following:


$$\begin{array}{c} {Adv}_{\overset{˚}{A}}^{\lambda_{\text{CSB}}}\left(p_{t}\right)\leq \frac{H_{n}^{2}}{\left|\mu \right|}+2({Adv}_{\overset{˚}{A}}^{\omega }(p_{t})) \end{array}$$ (10)

Since both $\frac{H_{n}^{2}}{\left|\mu \right|}$ and ${Adv}_{\overset{˚}{A}}^{\omega }(p_{t})$ are both infinitesimal, it follows that ${Adv}_{\overset{˚}{A}}^{\lambda_{CSB}}\left(p_{t}\right)$ is also infinitesimal in polynomial time *p*_t_. This effectively completes the proof of *Hypothesis 1*.

### 4.2 Informal security analysis

In this sub-section, we formulate and proof a number of theorems with the aim of demonstrating that our protocol is secure under all the adversarial capabilities in the Canetti–Krawczyk threat model.

**Theorem 1: Ephemeral secret leakage attacks are prevented**

**Proof:** During the mutual authentication between *SM*_j_ and *SP*_j_, the *SM*_j_ derives the session key as *ɸ*_SM_ =  *h* (*A*_3_||ℤ_1_||*T*_4_||*T*_6_) that it shares with *SP*_j_. Here, *A*_3_ =  *A*_1_.*h* (ℝ_2_||*TID*_SM_||*PID*_SM_||*SK*_SM_||*T*_6_) and ℤ_1_ =  *h* (**ℝ**_1_||*TID*_SP_||*SK*_SP_||) +  *h*(*PK*_SP_||*PK*_SM_||*PK*_CS_||*T*_4_) * *SK*_SP_ (mod *q*). Similarly, *SP*_j_ computes the session key as *ɸ*_SP_ =  *h* (*A*_4_||ℤ_1_||*T*_4_||*T*_6_), where *A*_4_ =  *A*_2_.*h* (ℝ_1_||*TID*_SP_||*PID*_SP_||*SK*_SP_||*T*_4_) and ℤ_1_ =  *h* (**ℝ**_1_||*TID*_SP_||*SK*_SP_||) +  *h*(*PK*_SP_||*PK*_SM_||*PK*_CS_||*T*_4_) * *SK*_SP_ (mod *q*). The derivation of both *A*_3_ and *A*_4_ incorporates short term secrets such as random nonces ℝ_2_ and ℝ_1_ respectively. In addition, long term secrets such as private keys (*SK*_SM_ and *SK*_SP_) for *SM*_j_ and *SP*_j_ are incorporated. As such, the adversary *Å* can only derive valid session keys when in possession of both long term and short term secrets (ephemerals) of *SM*_j_ and *SP*_j_. Since authentication messages *AM*_1_ =  {*TID*_SP_, *A*_1,_ ℤ_1,_ *T*_4_} and *AM*_2_ =  {*TID*_SP_^ * ^, *A*_2_, ℤ_2_, *T*_6_} exchanged during mutual authentication do not contain these parameters in plaintext, *Å* cannot access them. As such, adversarial derivation of valid session keys flops.

**Theorem 2: Backward and forward key secrecy is preserved**

**Proof:** In the proposed protocol, four session keys are derived. During *SM*_j_ ↔  *SP*_j_ authentication, *SM*_j_ derives the session key as *ɸ*_SM_ =  *h* (*A*_3_||ℤ_1_||*T*_4_||*T*_6_) while *SP*_j_ computes the session key as *ɸ*_SP_ =  *h* (*A*_4_||ℤ_1_||*T*_4_||*T*_6_). Similarly, during *CS*_i_ ↔  *SM*_j_, the *CS*_i_ derives session key *ɸ*_SC_ =  *h* (*B*_3_|| *f* (*PID*_SM_, *PID*_CS_)||ℂ_SM_||ℂ_CS_) while the *SM*_j_ calculates session key *ɸ*_CM_ =  *h* (*B*_4_||*f* (*PID*_CS_, *PID*_SM_)||ℂ_SM_ ||ℂ_CS_). Here, *A*_3_ =  *A*_1_.*h* (ℝ_2_||*TID*_SM_||*PID*_SM_||*SK*_SM_||*T*_6_), *A*_1_ =  *G*.*h* (**ℝ**_1_||*TID*_SP_||*PID*_SP_||*SK*_SP_||*T*_4_), ℤ_1_ =  *h* (**ℝ**_1_||*TID*_SP_||*SK*_SP_||) +  *h*(*PK*_SP_||*PK*_SM_||*PK*_CS_||*T*_4_) * *SK*_SP_ (mod *q*), *A*_4_ =  *A*_2_.*h* (ℝ_1_||*TID*_SP_||*PID*_SP_||*SK*_SP_||*T*_4_), *A*_2_ =  *G.h* (ℝ_2_||*TID*_SM_||*PID*_SM_||*SK*_SM_||*T*_6_), *B*_3_ =  *B*_1_. h (**ℝ**_4_||*SK*_SM_||*PID*_SM_||*T*_10_), ℂ_SM_ =  *η*_2_ +  *h*(*PID*_SM_||*ID*_RA_||*P*_SM_||*PK*_RA_) * *MK*_RA_ (mod *q*), ℂ_CS_ =  *η*_1_ +  *h*(*PID*_CS_||*ID*_RA_||*P*_CS_||*PK*_RA_) * *MK*_RA_ (mod *q*) and *B*_4_ =  *B*_2_. *h* (**ℝ**_3_||*SK*_CS_||*PID*_CS_||*T*_8_). Evidently, all these session keys incorporate random nonces. These random nonces are independently derived by each of the communication entities and are never shared in plaintext over public channels. As such, even if *Å* captures the current session keys, adversarial derivation of session keys for past and subsequent communication session based on these keys will fail.

**Theorem 3: Eavesdropping and session hijacking attacks are thwarted**

**Proof:** Suppose that *Å* is interested in hijacking the communication session so as to convince unsuspecting network entities that they are communicating with legitimate entity. Therefore, an attempt is made to eavesdrop the communication channel for ephemerals that may facilitate the derivation of valid session keys. During *SM*_j_ ↔  *SP*_j_ authentication, messages *AM*_1_ =  {*TID*_SP_, *A*_1,_ ℤ_1,_ *T*_4_} and *AM*_2_ =  {*TID*_SP_^ * ^, *A*_2_, ℤ_2_, *T*_6_} are exchanged. Here, *A*_1_ =  *G*.*h* (**ℝ**_1_||*TID*_SP_||*PID*_SP_||*SK*_SP_||*T*_4_), ℤ_1_ =  *h* (**ℝ**_1_||*TID*_SP_||*SK*_SP_||) +  *h*(*PK*_SP_||*PK*_SM_||*PK*_CS_||*T*_4_) * *SK*_SP_ (mod *q*), *A*_2_ =  *G.h* (ℝ_2_||*TID*_SM_||*PID*_SM_||*SK*_SM_||*T*_6_) and ℤ_2_ =  *h* (ℝ_2_||*TID*_SM_||*PID*_SM_||*SK*_SM_||*T*_6_) + *h*(*ɸ*_SM_||*PK*_SP_||*PK*_CS_||*T*_6_) * *SK*_SM_ (mod *q*). Next, adversarial derivation of *ɸ*_SM_ =  *h* (*A*_3_||ℤ_1_||*T*_4_||*T*_6_) and *ɸ*_SP_ =  *h* (*A*_4_||ℤ_1_||*T*_4_||*T*_6_) is attempted. Although timestamp *T*_4_ and *T*_6_ as well as signature ℤ_1_ may be obtained from the eavesdropped messages, the attacker still needs parameters *A*_3_ and *A*_4_ to successfully derive the much needed session keys *ɸ*_SM_ and *ɸ*_SP_. While *A*_3_ is independently calculated at the *SM*_j_, *A*_4_ is independently derived at the *SP*_j_. Since these two values are never transmitted in messages *AM*_1_ and *AM*_2_ and hence cannot be eavesdropped, these attacks fail. Similarly, messages *KM*_1_ =  {*TID*_SM_, *B*_1_, ℂ_CS_, ℤ_3_, *PID*_CS_^ * ^, *T*_8_} and *KM*_2_ =  {*TID*_SM_^ * ^, ℂ_SM_, *B*_2_, ℤ_4_, *T*_10_} are exchanged during *CS*_i_ ↔  *SM*_j_ authentication. Here, *B*_1_ =  *G*.*h* (**ℝ**_3_||*SK*_CS_||*PID*_CS_||*T*_8_), ℂ_CS_ =  *η*_1_ +  *h*(*PID*_CS_||*ID*_RA_||*P*_CS_||*PK*_RA_) * *MK*_RA_ (mod *q*), ℤ_3_ =  *h* (**ℝ**_3_||*SK*_CS_||*PID*_CS_||*T*_8_) +  *h* (*PK*_CS_||ℂ_CS_||*PID*_CS_^*^||*TID*_SM_) * *SK*_CS_ (mod *q*), *PID*_CS_^ *^ =  *PID*_CS_^ ⊕ *h* (^*PID*_SM_||*ID*_RA_||*T*_8_^),^ ℂ_SM_ =  *η*_2_ +  *h*(*PID*_SM_||*ID*_RA_||*P*_SM_||*PK*_RA_) * *MK*_RA_ (mod *q*), *B*_2_ =  *G*.*h* (**ℝ**_4_||*SK*_SM_||*PID*_SM_||*T*_10_) and ℤ_4_ =  *h* (**ℝ**_4_||*SK*_SM_||*PID*_SM_||*T*_10_) +  *h* (*PK*_SM_||ℂ_SM_||*ID*_RA_||*ɸ*_SC_) * *SK*_SM_ (mod *q*). To derive session keys *ɸ*_CM_ =  *h* (*B*_4_||*f* (*PID*_CS_, *PID*_SM_)||ℂ_SM_ ||ℂ_CS_) and *ɸ*_SC_ =  *h* (*B*_3_|| *f* (*PID*_SM_, *PID*_CS_)||ℂ_SM_||ℂ_CS_), *Å* still needs *B*_4_, *PID*_SM_ and *B*_3_. Whereas *B*_4_ is derived at the *CS*_i_, *PID*_SM_ is generated at the RA while *B*_3_ is calculated at the *SM*_j_. Once again, these two attacks fail since these parameters cannot be eavesdropped from the exchanged messages.

**Theorem 4: Our scheme offers anonymity and untraceability**

**Proof:** The aim of the adversary here is to listen to the communication channel with the aim of associating the communication sessions to particular network entities. As already demonstrated, messages *AM*_1_, *AM*_2_, *KM*_1_ and *KM*_2_ are exchanged over the public channels. Here, *AM*_1_ =  {*TID*_SP_, *A*_1,_ ℤ_1,_ *T*_4_}, *AM*_2_ =  {*TID*_SP_^ * ^, *A*_2_, ℤ_2_, *T*_6_}, *KM*_1_ =  {*TID*_SM_, *B*_1_, ℂ_CS_, ℤ_3_, *PID*_CS_^ * ^, *T*_8_} and *KM*_2_ =  {*TID*_SM_^ * ^, ℂ_SM_, *B*_2_, ℤ_4_, *T*_10_}. Evidently, real identities of the communicating entities are not included in these messages. As such, only transient identities for *SP*_j_ (*TID*_SP_) and *SM*_j_ (*TID*_SM_) as well as the pseudo-identity of *CS*_i_ (*PID*_CS_^*^) can be deciphered. Therefore, these messages cannot be linked to any communicating entities. Random nonces are incorporated in all these messages since *A*_1_ =  *G*.*h* (**ℝ**_1_||*TID*_SP_||*PID*_SP_||*SK*_SP_||*T*_4_), ℤ_1_ =  *h* (**ℝ**_1_||*TID*_SP_||*SK*_SP_||) +  *h*(*PK*_SP_||*PK*_SM_||*PK*_CS_||*T*_4_) * *SK*_SP_ (mod *q*), *A*_2_ =  *G.h* (ℝ_2_||*TID*_SM_||*PID*_SM_||*SK*_SM_||*T*_6_), ℤ_2_ =  *h* (ℝ_2_||*TID*_SM_||*PID*_SM_||*SK*_SM_||*T*_6_) + *h*(*ɸ*_SM_||*PK*_SP_||*PK*_CS_||*T*_6_) * *SK*_SM_ (mod *q*), *B*_1_ =  *G*.*h* (**ℝ**_3_||*SK*_CS_||*PID*_CS_||*T*_8_) and *B*_2_ =  *G*.*h* (**ℝ**_4_||*SK*_SM_||*PID*_SM_||*T*_10_). In addition, timestamps are also part of all the exchanged messages. As such, all the exchanged messages are always unique for each session and hence cannot be easily associated to the communicating parties.

**Theorem 5:** **Known session-specific temporary information (KSSTI) are prevented**

**Proof:** In our scheme, the session keys are computed using a number of short term and long term keys. These session keys include *ɸ*_SM_ =  *h* (*A*_3_||ℤ_1_||*T*_4_||*T*_6_), *ɸ*_SP_ =  *h* (*A*_4_||ℤ_1_||*T*_4_||*T*_6_), *ɸ*_CM_ =  *h* (*B*_4_||*f* (*PID*_CS_, *PID*_SM_)||ℂ_SM_ ||ℂ_CS_) and *ɸ*_SC_ =  *h* (*B*_3_|| *f* (*PID*_SM_, *PID*_CS_)||ℂ_SM_||ℂ_CS_). Here, *A*_3_ =  *A*_1_.*h* (ℝ_2_||*TID*_SM_||*PID*_SM_||*SK*_SM_||*T*_6_), *A*_1_ =  *G*.*h* (**ℝ**_1_||*TID*_SP_||*PID*_SP_||*SK*_SP_||*T*_4_), ℤ_1_ =  *h* (**ℝ**_1_||*TID*_SP_||*SK*_SP_||) +  *h*(*PK*_SP_||*PK*_SM_||*PK*_CS_||*T*_4_) * *SK*_SP_ (mod *q*), *A*_4_ =  *A*_2_.*h* (ℝ_1_||*TID*_SP_||*PID*_SP_||*SK*_SP_||*T*_4_), *A*_2_ =  *G.h* (ℝ_2_||*TID*_SM_||*PID*_SM_||*SK*_SM_||*T*_6_), *B*_3_ =  *B*_1_.*h* (**ℝ**_4_||*SK*_SM_||*PID*_SM_||*T*_10_), ℂ_SM_ =  *η*_2_ +  *h*(*PID*_SM_||*ID*_RA_||*P*_SM_||*PK*_RA_) * *MK*_RA_ (mod *q*), ℂ_CS_ =  *η*_1_ +  *h*(*PID*_CS_||*ID*_RA_||*P*_CS_||*PK*_RA_) * *MK*_RA_ (mod *q*) and *B*_4_ =  *B*_2_.*h* (**ℝ**_3_||*SK*_CS_||*PID*_CS_||*T*_8_). The short term keys are exampled by random nonces such as **ℝ**_1_, ℝ_2_, **ℝ**_3_ and **ℝ**_4_. On the other hand, long term keys include secret keys such as *SK*_SM_, *SK*_SP_, *PK*_SP_, *PK*_SM_, *PK*_CS_, *PK*_RA_ and *MK*_RA_. As such, the loss of session specific ephemerals such as short term keys does not enable the attacker to compromise the session keys.

**Theorem 6: Our protocol is resilient against message replay attacks**

**Proof:** To prevent this attack, timestamps and random nonces are incorporated in all the exchanged messages during the mutual authentication phase. For instance, messages *AM*_1_ =  {*TID*_SP_, *A*_1,_ ℤ_1,_ *T*_4_}, *AM*_2_ =  {*TID*_SP_^ * ^, *A*_2_, ℤ_2_, *T*_6_}, *KM*_1_ =  {*TID*_SM_, *B*_1_, ℂ_CS_, ℤ_3_, *PID*_CS_^ * ^, *T*_8_} and *KM*_2_ =  {*TID*_SM_^ * ^, ℂ_SM_, *B*_2_, ℤ_4_, *T*_10_} all contain timestamps. On the other hand, ephemerals *A*_1_ =  *G*.*h* (**ℝ**_1_||*TID*_SP_||*PID*_SP_||*SK*_SP_||*T*_4_), ℤ_1_ =  *h* (**ℝ**_1_||*TID*_SP_||*SK*_SP_||) +  *h*(*PK*_SP_||*PK*_SM_||*PK*_CS_||*T*_4_) * *SK*_SP_ (mod *q*), *A*_2_ =  *G.h* (ℝ_2_||*TID*_SM_||*PID*_SM_||*SK*_SM_||*T*_6_), ℤ_2_ =  *h* (ℝ_2_||*TID*_SM_||*PID*_SM_||*SK*_SM_||*T*_6_) + *h*(*ɸ*_SM_||*PK*_SP_||*PK*_CS_||*T*_6_) * *SK*_SM_ (mod *q*), *B*_1_ =  *G*.*h* (**ℝ**_3_||*SK*_CS_||*PID*_CS_||*T*_8_) and *B*_2_ =  *G*.*h* (**ℝ**_4_||*SK*_SM_||*PID*_SM_||*T*_10_) all incorporate random nonces in their derivations. Any replays of old messages will be easily detected by the timestamp checks and the sessions will only continue provided that | *T*_5_- *T*_4_ | < *∆ T*, | *T*_7_- *T*_6_ | < *∆ T*, | *T*_9_- *T*_8_ | < *∆ T* and | *T*_11_- *T*_10_ | < *∆ T*. Otherwise, the sessions will be aborted in all the instances.

**Theorem 7: Strong mutual authentication is achieved**

**Proof:** In our scheme, all the exchanged messages after the registration phase are mutually verified by the receivers. For instance, on receiving *AM*_1_ =  {*TID*_SP_, *A*_1,_ ℤ_1,_ *T*_4_}, *SM*_j_ validates it by checking if | *T*_5_- *T*_4_ | < *∆ T* and ℤ_1_.*G* ≟  *A*_1_ +  *h*(*PK*_SP_||*PK*_SM_||*PK*_CS_||*T*_4_) * *PK*_SP_. On the other hand, upon receiving message *AM*_2_ =  {*TID*_SP_^ * ^, *A*_2_, ℤ_2_, *T*_6_}, the *SP*_j_ checks if | *T*_7_- *T*_6_ | < *∆ T* and ℤ_2_.*G* ≟  *A*_2_ +  *h*(*ɸ*_SP_||*PK*_SP_||*PK*_CS_||*T*_6_). Similarly, after getting message *KM*_1_ =  {*TID*_SM_, *B*_1_, ℂ_CS_, ℤ_3_, *PID*_CS_^ * ^, *T*_8_}, *SM*_j_ confirms whether | *T*_9_- *T*_8_ | < *∆ T*, ℂ_CS_.*G* ≟  *P*_CS_ +  *h*(*PID*_CS_||*ID*_RA_||*P*_CS_||*PK*_RA_) * *PK*_RA_ and ℤ_3_.*G* ≟  *B*_1_ +  *h* (*PK*_CS_||ℂ_CS_||*PID*_CS_^*^||*TID*_SM_) * *PK*_CS_. On the other hand, upon receiving message ^*KM*2 =  {^*TID*_SM_^ * ^, ℂ_SM_, *B*_2_, ℤ_4_, *T*_10_^}, the^ *CS*_i_ checks if | *T*_11_- *T*_10_ | < *∆ T*, ℂ_SM_.*G* ≟  *P*_SM_ +  *h*(*PID*_SM_||*ID*_RA_||*P*_SM_||*PK*_RA_) * *PK*_RA_ and ℤ_4_.*G* ≟ *B*_2_ +  *h* (*PK*_SM_||ℂ_SM_||*ID*_RA_||*ɸ*_CM_) * *PK*_SM_. In all these verification instances, the sessions are terminated upon checks failure.

**Theorem 8: Session keys are negotiated**

**Proof:** Immediately after successful mutual authentication, all the interacting parties derive the shared session keys for traffic protection. For instance, after authenticating *SP*_j_, the *SM*_j_ derives session key as *ɸ*_SM_ =  *h* (*A*_3_||ℤ_1_||*T*_4_||*T*_6_). On the other hand, after verifying *SM*_j_, the *SP*_j_ computes the session keys as *ɸ*_SP_ =  *h* (*A*_4_||ℤ_1_||*T*_4_||*T*_6_). Similarly, session key *ɸ*_SC_ =  *h* (*B*_3_|| *f* (*PID*_SM_, *PID*_CS_)||ℂ_SM_||ℂ_CS_) is calculated by *SM*_j_ upon validation of the *CS*_i_. In the same manner, session key *ɸ*_CM_ =  *h* (*B*_4_||*f* (*PID*_CS_, *PID*_SM_)||ℂ_SM_ ||ℂ_CS_) is derived by the *CS*_i_ upon verification of *SM*_j_.

**Theorem 9: All the blocks are validated before addition to the blockchain**

**Proof:** In the proposed protocol, three-level validation is executed on all the blocks before their addition to the blockchain. Suppose that a verifier *ℽ* is interested in verifying block *β*_n_ stored in a given blockchain. To accomplish this, *ℽ* derives ℜ^ *^ on all enciphered transactions in *β*_n_. In addition, it computes the ℍ_C_^ *^ on *β*_n_. Thereafter, it verifies whether ℜ^*^≟ ℜ as well as ℍ_C_^*^≟ ℍ_C_. Basically, block *β*_n_ is rejected when these two validations flop. However, if the verifications are successful, *ℽ* proceeds to validate signature $E_{{sig}_{\beta_{n}}}$on these transactions using *E*_ver_. Since *β*_n_ incorporates ℍ_P_ (hash value of the preceding block), it is infeasible for *Å* to modify or corrupt the information stored in *β*_n_.

**Theorem 10: Forgery and MitM attacks are prevented**

**Proof:** The aim of these attacks is for *Å* to intercept exchanged messages and modify them to fool other network entities. Suppose that *Å* has captured authentication message *AM*_1_ =  {*TID*_SP_, *A*_1,_ ℤ_1,_ *T*_4_} and wants to generate bogus message *AM*_1_^Å^. Here, *A*_1_ =  *G*.*h* (**ℝ**_1_||*TID*_SP_||*PID*_SP_||*SK*_SP_||*T*_4_), ℤ_1_ =  *h* (**ℝ**_1_||*TID*_SP_||*SK*_SP_||) +  *h*(*PK*_SP_||*PK*_SM_||*PK*_CS_||*T*_4_) * *SK*_SP_ (mod *q*). Evidently, *Å* requires private tokens such as *SK*_SP_ and *PID*_SP_ as well as timestamp *T*_4_ to derive parameters *A*_1_^Å^ and ℤ_1_^Å^. Suppose that *Å* is interested in the derivation of message *AM*_2_ =  {*TID*_SP_^ * ^, *A*_2_, ℤ_2_, *T*_6_}, where *A*_2_ =  *G.h* (ℝ_2_||*TID*_SM_||*PID*_SM_||*SK*_SM_||*T*_6_) and ℤ_2_ =  *h* (ℝ_2_||*TID*_SM_||*PID*_SM_||*SK*_SM_||*T*_6_) + *h*(*ɸ*_SM_||*PK*_SP_||*PK*_CS_||*T*_6_) * *SK*_SM_ (mod *q*). Clearly, this requires timestamp *T*_6_ and secret tokens *SK*_SM_ and *PID*_SM_. Similarly, *KM*_1_ =  {*TID*_SM_, *B*_1_, ℂ_CS_, ℤ_3_, *PID*_CS_^ * ^, *T*_8_} and *KM*_2_ =  {*TID*_SM_^ * ^, ℂ_SM_, *B*_2_, ℤ_4_, *T*_10_} derivation requires secrets *SK*_CS_, *PID*_CS_, *SK*_SM_, *PID*_SM_ as well as timestamps *T*_8_ and *T*_10_. This is because *B*_1_ =  *G*.*h* (**ℝ**_3_||*SK*_CS_||*PID*_CS_||*T*_8_), ℂ_CS_ =  *η*_1_ +  *h*(*PID*_CS_||*ID*_RA_||*P*_CS_||*PK*_RA_) * *MK*_RA_ (mod *q*), ℤ_3_ =  *h* (**ℝ**_3_||*SK*_CS_||*PID*_CS_||*T*_8_) +  *h* (*PK*_CS_||ℂ_CS_||*PID*_CS_^*^||*TID*_SM_) * *SK*_CS_ (mod *q*), *PID*_CS_^ *^ =  *PID*_CS_^ ⊕ *h* (^*PID*_SM_||*ID*_RA_||*T*_8_^),^ ℂ_SM_ =  *η*_2_ +  *h*(*PID*_SM_||*ID*_RA_||*P*_SM_||*PK*_RA_) * *MK*_RA_ (mod *q*), *B*_2_ =  *G*.*h* (**ℝ**_4_||*SK*_SM_||*PID*_SM_||*T*_10_) and ℤ_4_ =  *h* (**ℝ**_4_||*SK*_SM_||*PID*_SM_||*T*_10_) +  *h* (*PK*_SM_||ℂ_SM_||*ID*_RA_||*ɸ*_SC_) * *SK*_SM_ (mod *q*). As such, forgery and MitM attacks are thwarted.

**Theorem 11: This protocol is robust against privileged insider attacks**

**Proof:** Suppose that the RA wants to obtain secret values for the *SM*_j_, *SP*_j_ and *CS*_i_. However, none of these devices generate and submit their secret parameters to the RA. On the contrary, it is the RA that creates these security tokens including secret keys. However, the RA erases these secret keys upon sending registration messages to the recipients. For instance, after sending registration message *R*_1_ =  {*ID*_RA_, ℂ_CS_, *PID*_CS_, *f* (*PID*_CS_, *d*)} to *CS*_i_ over secure channels, RA erases random secret key *η*_1_ used to derive some of these parameters. Similarly, after sending registration message *R*_2_ =  {*TID*_SM_, *PID*_SM_} to *CS*_i_ over secure channels, the RA erases random secret key *η*_2_. Therefore, privileged insiders at the RA are unable to access these secret values that may enable them derive ephemerals for the network entities.

**Theorem 12: Physical and side-channeling attacks are prevented**

**Proof:** The assumption made in this attack is that *Å* can physically capture *SM*_j_ as well as any other smart device within the smart grid system. In our protocol, the *SM*_j_ store parameter set {(*TID*_SM_, *PID*_SM_), ℂ_SM_, *f* (*PID*_SM_, *d*), *ID*_RA_, (*SK*_SM_, *PK*_SM_)} during the registration phase. Therefore, *Å* may opt to use power analysis to retrieve these parameters. Next, an attempt is made to utilize these parameters to derive session keys *ɸ*_SM_ =  *h* (*A*_3_||ℤ_1_||*T*_4_||*T*_6_) and *ɸ*_SC_ =  *h* (*B*_3_|| *f* (*PID*_SM_, *PID*_CS_)||ℂ_SM_||ℂ_CS_). Here, *A*_3_ =  *A*_1_.*h* (ℝ_2_||*TID*_SM_||*PID*_SM_||*SK*_SM_||*T*_6_), ℤ_1_ =  *h* (**ℝ**_1_||*TID*_SP_||*SK*_SP_||) +  *h*(*PK*_SP_||*PK*_SM_||*PK*_CS_||*T*_4_) * *SK*_SP_ (mod *q*), *B*_3_ =  *B*_1_.*h* (**ℝ**_4_||*SK*_SM_||*PID*_SM_||*T*_10_), ℂ_SM_ =  *η*_2_ +  *h*(*PID*_SM_||*ID*_RA_||*P*_SM_||*PK*_RA_) * *MK*_RA_ (mod *q*), ℂ_CS_ =  *η*_1_ +  *h*(*PID*_CS_||*ID*_RA_||*P*_CS_||*PK*_RA_) * *MK*_RA_ (mod *q*). Evidently, *Å* still requires random nonces **ℝ**_1_,ℝ_2_ and **ℝ**_4_, timestamps *T*_4_, *T*_6_, and *T*_10_, certificate ℂ_CS_, identity *ID*_RA_, long term key *MK*_RA_ and ephemerals such as *TID*_SP_, *SK*_SP_, *B*_1_, *η*_1_ and *η*_2_. Therefore, the communication process is still secure in the face of these two attacks.

**Theorem 13: This protocol can withstand impersonation attacks**

**Proof:** Suppose that adversary *Å* is interested in masquerading as legitimate *SP*_j_ with the intention of generating authentication message *AM*_1_ =  {*TID*_SP_, *A*_1,_ ℤ_1,_ *T*_4_}. Here, *A*_1_ =  *G*.*h* (**ℝ**_1_||*TID*_SP_||*PID*_SP_||*SK*_SP_||*T*_4_) and ℤ_1_ =  *h* (**ℝ**_1_||*TID*_SP_||*SK*_SP_||) +  *h*(*PK*_SP_||*PK*_SM_||*PK*_CS_||*T*_4_) * *SK*_SP_ (mod *q*). Let us assume that *Å* has generated bogus timestamp *T*_4_^ *^ and random secret **ℝ**_1_^ *^ and hence wants to compute legitimate *A*_1_^ *^ and signature ℤ_1_^ * ^. However, devoid of valid secret parameters *SK*_SP_ and *PID*_SP_, it is infeasible for *Å* to succeed in these derivations. Therefore, the construction of message *AM*_1_ =  {*TID*_SP_, *A*_1,_ ℤ_1,_ *T*_4_} flops. Suppose that *Å* wants to masquerade as smart meter *SM*_j_ by attempting to generate message *AM*_2_ =  {*TID*_SP_^ * ^, *A*_2_, ℤ_2_, *T*_6_}. Here, *A*_2_ =  *G.h* (ℝ_2_||*TID*_SM_||*PID*_SM_||*SK*_SM_||*T*_6_) and ℤ_2_ =  *h* (ℝ_2_||*TID*_SM_||*PID*_SM_||*SK*_SM_||*T*_6_) + *h*(*ɸ*_SM_||*PK*_SP_||*PK*_CS_||*T*_6_) * *SK*_SM_ (mod *q*). Once again, this impersonation will flop if *Å* cannot access secret parameters *PID*_SM_ and *SK*_SM_.

**Theorem 14: This protocol eliminates key escrow issues**

**Proof:** During the registration phase, the *SM*_j_ store parameter set {(*TID*_SM_, *PID*_SM_), ℂ_SM_, *f* (*PID*_SM_, *d*), *ID*_RA_, (*SK*_SM_, *PK*_SM_)}. On the other hand, the RA secretly stores *MK*_RA_ as well as parameter set {(*TID*_SP_, *PID*_SP_), (*SK*_SP_, *PK*_SP_)}. Similarly, *CS*_i_ stores parameter set {*ID*_RA_, ℂ_CS_, *PID*_CS_, *f*(*PID*_CS_, *d*), (*SK*_CS_, *PK*_CS_)}. During key management, mutual authentication and key negotiation phases, our scheme does not need any verifier tables. Instead, all the required parameters are independently derived and validated by the *SM*_j_, *CS*_i_ and *SP*_j_.

## 5. Performance evaluation

In authentication protocols, computation costs, supported security features and communication costs are the most widely deployed performance metrics during their performance evaluations. Therefore, these three metrics are deployed in this section to appraise the proposed protocol. In addition, we describe the blockchain implementation of our protocol. Moreover, comparative evaluations of this scheme are provided against other related protocols as described in the sub-sections below.

### 5.1 Computation complexities

In this sub-section, the computation overheads are derived for both *SP*_j_ and *SM*_j_ authentication. The *SP*_j_ and *CS*_i_ experimentations are executed on a machine running Windows 10 Pro 64 bit operating system on Intel(R) Core i5-2310M processor, installed with 2 GB of RAM, and with 3 GHz Clock frequency. On the other hand, the *SM*_j_ experimentations are run on Raspberry Pi-3 quad-core, installed with a 1.2 GHz CPU and 1GB of RAM. To execute the various cryptographic primitives, the MIRACL Cryptographic library is deployed. Under these specifications, the notations and execution durations for the various cryptographic primitives are presented in Table 4.

**Table 4:** Execution time for various cryptographic operations.

Cryptographic primitive	Notation	*SP*_j_/*CS*_i_ (ms)	*SM*_j_ (ms)
One-way hashing operation	*T*_H_	0.0046	0.0406
Elliptic curve point multiplication	*T*_ECM_	1.8212	6.9780
Elliptic curve point addition	*T*_ECA_	0.0075	0.1436
Bilinear pairing	*T*_BP_	8.4681	51.6620
Modular exponentiation	*T*_E_	0.0862	0.5310
Modular multiplication	*T*_M_	0.0628	0.4768
Symmetric encryption/decryption	*T*_SE_	0.0035	0.0957
Map to point hash	*T*_MH_	13.206	78.2710

During the *SM*_j_ ↔  *SP*_j_ authentication, 11*T*_H_, 8*T*_ECM_ and 2*T*_ECA_ operations are executed. Here, the *SM*_j_ executes 5 *T*_H_ +  3*T*_ECM_ +  *T*_ECA_ while the *SP*_j_ executes 6 *T*_H_ +  5*T*_ECM_ +  *T*_ECA_ operations. On the other hand, using the cryptographic run-times in Table 4 above, the computation complexity of the proposed protocol is detailed in Table 5. In addition, the computation complexities of other related schemes is also elaborated.

**Table 5:** Computation complexities comparisons.

Scheme	*SM*_j_	*SP*_j_/ Utility control	Total costs (ms)
Tsai & Lo [21]	4*T*_ECM_ + 5*T*_H_+ *T*_E_ + *T*_ECA_	3 *T*_ECM_ + 5*T*_H_+ *T*_E_ +2*T*_BP_+ *T*_ECA_	51.3061
Saxena et al. [40]	4*T*_ECM_ + 2*T*_H_+ *T*_SE_+*T*_BP_	4*T*_ECM_ + 2*T*_H_	87.0449
Mahmood et al. [47]	3*T*_ECM_ + 5*T*_H_ + *T*_SE_ +*T*_BP_ +2*T*_MH_	3*T*_ECM_ + 5*T*_H_ + *T*_SE_ +*T*_BP_ +2*T*_MH_+*T*_E_	269.8931
Odelu et al. [55]	3*T*_ECM_ + 6*T*_H_+ *T*_E_+ 3*T*_ECA_	2*T*_ECM_ + 6*T*_H_+ *T*_E_+2*T*_BP_+ 3*T*_ECA_	42.8543
He et al. [56]	4*T*_ECM_ + 5*T*_H_+ *T*_ECA_	6*T*_ECM_ + 6*T*_H_+ 2*T*_ECA_	39.2284
Khan et al. [57]	4*T*_ECM_ +10*T*_H_ +11 *T*_SE_	4*T*_ECM_ +9*T*_H_ +11 *T*_SE_	36.7354
Proposed	3*T*_ECM_ + 5 *T*_H_ + *T*_ECA_	5*T*_ECM_ + 6 *T*_H_ + *T*_ECA_	30.4217

**Fig 9:**
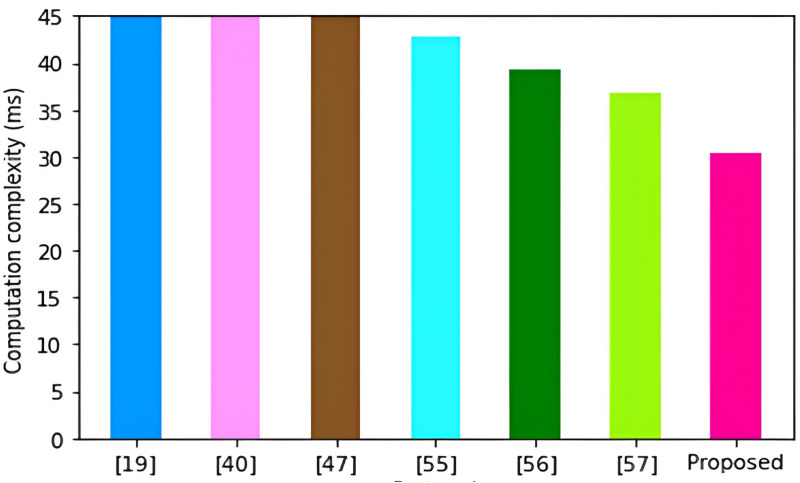
Computation complexities.

As shown in Fig 9, the protocol in [47] incurs the highest computation complexity of 269.8931ms. These high complexities can be explained by the high number of bilinear pairing operations and point multiplications that are executed in this scheme.

This is followed by the schemes in [19,40,55–57] with computation complexities of 87.0449 ms, 51.3061 ms, 42.8543 ms, 39.2284 ms and 36.7354 ms respectively. On the other hand, the proposed protocol incurs the least computation complexity of only 30.4217 ms. This is because our protocol majorly executes one-way hashing operations and a few point multiplication operations.

### 5.2 Communication complexities

In this section, the number and size of the messages exchanged during the mutual authentication phase, as well as the key management phase are taken into consideration. For mutual authentication, messages *AM*_1_ =  {*TID*_SP_, *A*_1,_ ℤ_1,_ *T*_4_} and *AM*_2_ =  {*TID*_SP_^ * ^, *A*_2_, ℤ_2_, *T*_6_} are exchanged. Here, *A*_1_ =  *G*.*h* (**ℝ**_1_||*TID*_SP_||*PID*_SP_||*SK*_SP_||*T*_4_), ℤ_1_ =  *h* (**ℝ**_1_||*TID*_SP_||*SK*_SP_||) +  *h*(*PK*_SP_||*PK*_SM_||*PK*_CS_||*T*_4_) * *SK*_SP_ (mod *q*), *A*_2_ =  *G.h* (ℝ_2_||*TID*_SM_||*PID*_SM_||*SK*_SM_||*T*_6_) and ℤ_2_ =  *h* (ℝ_2_||*TID*_SM_||*PID*_SM_||*SK*_SM_||*T*_6_) + *h*(*ɸ*_SM_||*PK*_SP_||*PK*_CS_||*T*_6_) * *SK*_SM_ (mod *q*). Using the values in [6], Table 6 presents the sizes of the various parameters used in the proposed protocol as well as in other related schemes.

**Table 6:** Parameter sizes.

Operation	Size (bits)
Timestamp	32
Nonces	160
Temporary identifies	160
Public & secret keys	320
Certificate	160
Elliptic curve point	320
Signature	160
One way hashing	160

Using the values in Table 6 above, the derivation of the communication complexities of our protocol is detailed in Table 7 below.

**Table 7:** Derivation of communication complexity.

Message	Size (bits)
*AM*_1_ = {*TID*_SP_, *A*_1,_ ℤ_1,_ *T*_4_} *TID*_SP_ = ℤ_1_ = 160; *A*_1_=320; *T*_4_ = 32	672
*AM*_2_ = {*TID*_SP_*, *A*_2_, ℤ_2_, *T*_6_} *TID*_SP_*= ℤ_2_ = 160; *A*_2_=320; *T*_6_ = 32	672
Total	1344

Based on the derivations in Table 7 above, the communication complexity of the proposed protocol is 1344 bits. Table 8 presents the communication complexities of other related schemes.

**Table 8:** Communication complexities comparisons.

Scheme	Messages exchanged	Size (bits)
Tsai & Lo [21]	3	3424
Saxena et al. [40]	4	2627
Mahmood et al. [47]	3	1340
Odelu et al. [55]	3	1920
He et al. [56]	3	1632
Khan et al. [57]	2	3136
Proposed	2	1344

**Fig 10:**
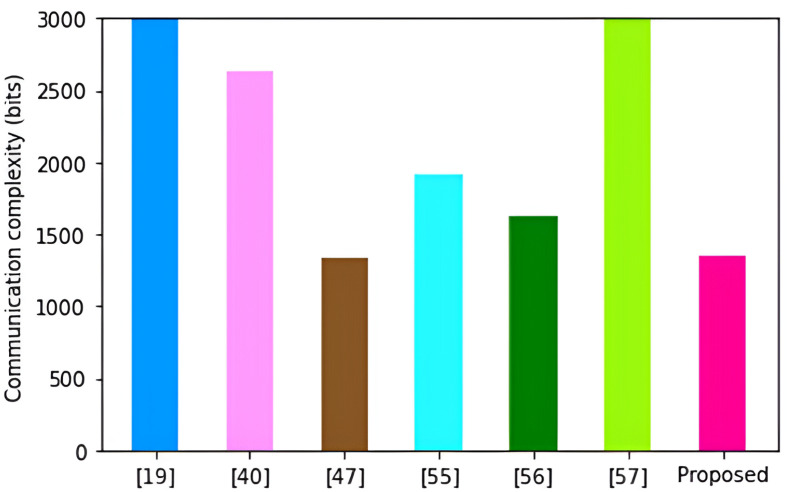
Communication complexities.

As shown in Fig 10, the protocol in [21] incurs the highest communication complexity of 3424 bits. This is followed by the schemes in [40,55–57], the proposed protocol and [47] with communication complexities of 3136 bits, 2627 bits, 1920 bits, 1632 bits, 1344 bits and 1340 bits respectively.

Although the protocol in [47] incurs the lowest communication costs, it has not been evaluated against threats such as side-channeling, physical, eavesdropping and session hijacking.

### 5.3 Supported security features

In this section, the attacks prevented and other features supported by the proposed protocol are compared with the ones offered by other related schemes. Table 9 presents these comparisons.

**Table 9:** Supported features comparisons.

	[21]	[40]	[55]	[56]	[47]	[57]	Proposed
Security features							
Key agreement	√	√	√	√	√	√	√
Mutual authentication	√	√	√	√	√	√	√
Backward key secrecy	√	×	√	√	√	√	√
Forward key secrecy	√	×	√	√	√	√	√
Anonymity	√	√	√	√	√	√	√
Non-traceability	×	×	×	√	√	√	√
Formal verification	√	√	√	√	√	√	√
Resilient against:							
Ephemeral secret leakage	×	×	×	×	×	×	√
Eavesdropping	×	√	×	×	×	×	√
Key escrow	×	×	×	×	×	×	√
Session hijacking	×	×	×	×	×	×	√
KSSTI	×	√	×	×	×	√	√
Replays	√	√	√	√	√	√	√
Forgery	×	√	×	√	×	×	√
MitM	√	√	√	√	√	√	√
Privileged insider	×	×	×	×	√	√	√
Physical	×	√	×	×	√	×	√
Side-channeling	×	×	×	×	×	√	√
Impersonation	√	√	√	√	√	√	√
		√ Supported; × Not supported or not considered					

**Fig 11:**
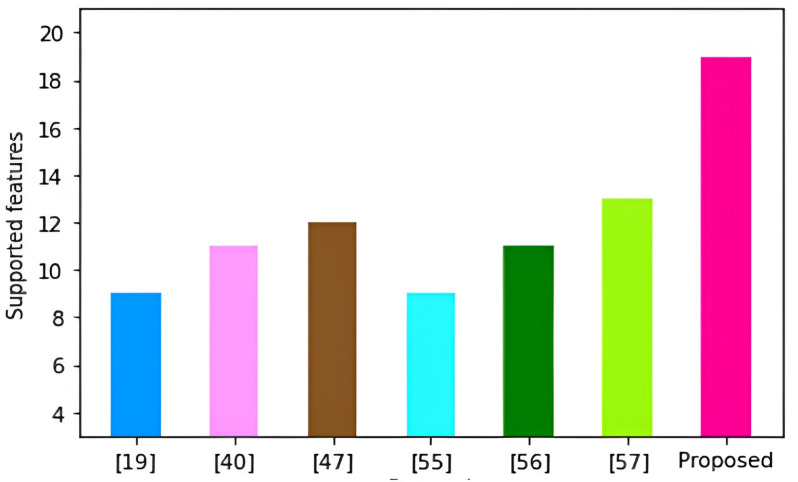
Supported functionalities.

As shown in Fig 11, the protocols in [21,55] support only 9 security features while the schemes in [40,56] support 11 features each. On the other hand, the schemes in [47,57] support 12 features and 13 features respectively. It is also evident that the proposed protocol offers support for all the 19 security features.

Using the scheme in [57] as the baseline, our scheme posts a 46.15% improvement in the supported security and privacy characteristics. Similarly, using the protocol in [57] as the baseline, our scheme posts a 17.19% reduction in the computation complexity. Considering smart grid components such as smart gas meters which are resource-limited, the proposed protocol is the most ideal for deployment in this environment.

### 5.4 Blockchain creation

To simulate the proposed protocol, we let *κ*_*τ*_ denote the transaction number threshold. When the number of transactions is equal to *κ*_*τ*_, the cloud servers within the network have to vote to select a new *C*_BL_ amongst themselves in a round-robin manner. This new *C*_BL_ is now in control of block *β*_n_ creation, validation and addition to BC in accordance with Algorithm 1. The Node.js was deployed as the scripting environment and the sizes of the various are presented in Table 10 below.

**Table 10:** Block *β*_n_ components size.

Component	Size (bits)
$V_{\beta_{n}}$	32
ℍ_P_	160
*ℜ*	160
*Ƭ*_stp_	32
${ID}_{C_{B}}$	160
*PK*_CS_	320
{$E_{{PK}_{CS}}\left(\Psi_{i}\right)|i=1,2,3,\ldots \kappa_{\tau }$)}	640
ℍ_C_	160
$E_{{sig}_{\beta_{n}}}$	160

Each of the generated transaction $\Psi_{k}$ is enciphered with the help of ECC and hence its output consists of two EC points. Consequently, each enciphered $\Psi_{k}$ is 640 bits in length and hence the total length of *β*_n_ is 1184 + 640*κ*_*τ*_ bits. During the PBFT based consensus algorithm voting process, the crash fault tolerance and Byzantine tolerance were 33% while *β*_n_ verification lasted between 60–70 transactions/ms. Taking *m* as the number of peer to peer nodes in the cloud servers, then *m*^*2*^ messages are exchange in each round of the four main phases of Algorithm 1. As such, the message complexity of this consensus algorithm is *O*(*m*^*2*^) and hence the cumulative volume of messages exchanged in this algorithm is given as 4*m*^*2*^ = *O*(*m*^*2*^). On the other hand, the computation complexity during the verification of *β*_n_ is 12*T*_ECM_ + 6*T*_H_ + 6*T*_ECA_, which is 21.927 ms. Similarly, the key management between any smart grid device and cloud server *CS*_i_ is 14*T*_H_ + 12*T*_ECM_ +  4*T*_ECA_ +  2*T*_PL_, which is 22.6288 ms.

**Fig 12:**
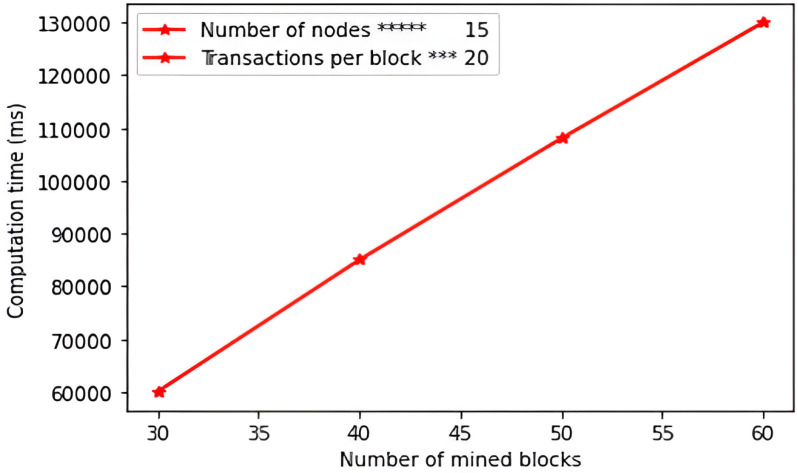
Effect of mined blocks on computation time.

We then investigate the effect of increasing the number of mined blocks *β*_ns_ on the computation complexity of the consensus algorithm. In this simulation, the number of transactions *κ*_*τ*_ per $\beta_{n_{i}}$ is kept constant. The results obtained are shown in Fig 12 below.

**Fig 13:**
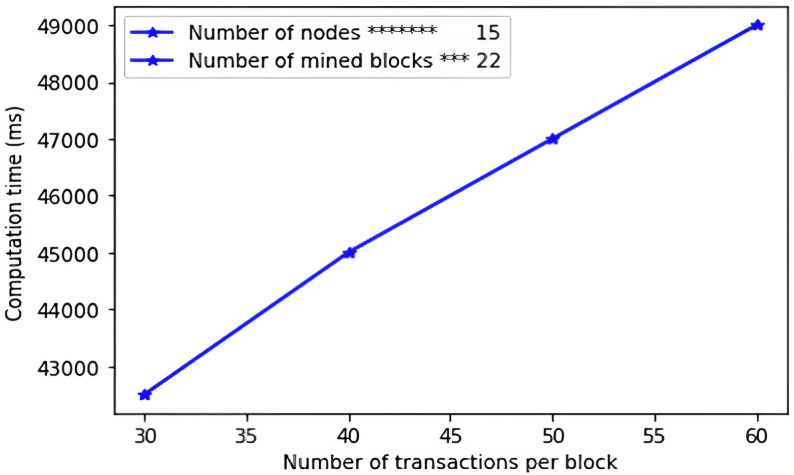
Effect of number of transactions on computation time.

As shown in Fig 12, there is a general increase in computation complexities upon increase in the number of $\beta_{n_{i}}$mined. Next, we investigate the effect of increasing the number of transactions *κ*_*τ*_ per $\beta_{n_{i}}$on the computation complexity of the consensus procedures. Here, we keep the number of mined *β*_ns_ constant for each chain. The results obtained are presented in Fig 13 below.

**Fig 14:**
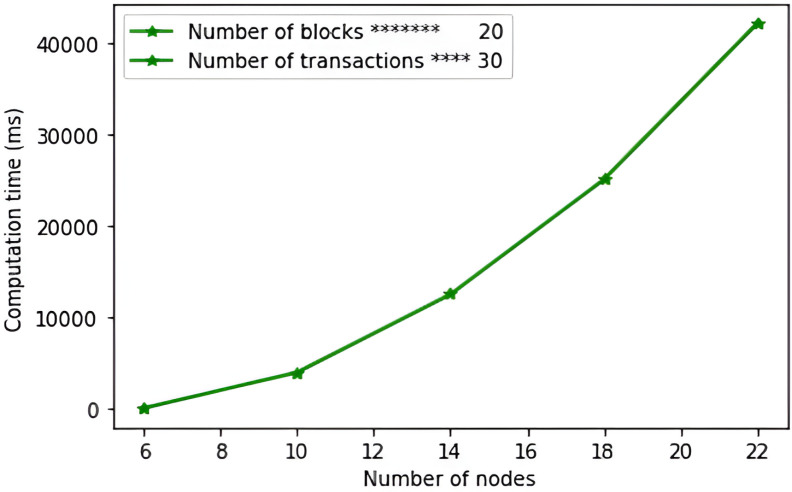
Effect of number of nodes on computation time.

It is evident from Fig 13 that as transactions *κ*_*τ*_ in $\beta_{n_{i}}$ surge, there is a corresponding increase in the computation complexities of the consensus procedures. Finally, we vary the number of nodes as the number of $\beta_{n_{i}}$and *κ*_*τ*_ are kept constant. Fig 14 shows the results obtained.

Based on the graph in Fig 14, it is clear that there is an exponential increase in computation complexity of the consensus procedures when the number of nodes is incremented. These increase in computation costs in all these three instances is attributed to the surging processing that must be accomplished during block generation, verification and addition to the blockchain.

## 6. Conclusion

Security and privacy issues in smart grids are serious challenges, owing to numerous vulnerabilities and threats that lurk in this environment. This has seen the development of numerous schemes to offer protection during message exchange between the smart meters and utility service providers. Majority of these security techniques are based on bilinear pairing operations and public key cryptography that are shown to incur heavy computation overheads. The frequent transmission of power consumption reports exposes these reports to security threats and results in high communication complexities. Therefore, an ideal authentication protocol has been developed in this paper to tackle these issues. Extensive security analysis has shown that it is provably secure under the ROR model. In addition, it has been shown to offers salient security and privacy features such as key agreement, mutual authentication, key secrecy, anonymity and untraceability. Moreover, it is resilient against eavesdropping, ephemeral secret leakage, key escrow, session hijacking, KSSTI, replays, forgery, MitM, privileged insider, physical, side-channeling and impersonation attacks. Therefore, it is demonstrated to be robust under all the adversarial capabilities in the Canetti–Krawczyk model. In terms of performance, it requires only 30.4217 ms computation costs, which is the lowest. Since it supports the highest number of security and privacy features, it is the most secure among its peers. Specifically, our protocol achieves a 17.19% reduction in the computation complexity and a 46.15% improvement in the supported security and privacy features. Future work lies in further reduction on the incurred communication complexities so that its efficiency can be reduced further.

## Appendix A: Data

**Table:** 

*ID*_RA_	1bc8e75c8ac2a1d7745bb6d338054b998b6b3f85
*G*	5bf0dd40718108af15d664d44e386f0f3e3becb8cb0280155f4023d56a207a4c05b00247ef0d2d1e
*MK*_RA_	ac92161fee6190c664294d4f7f011dca76ed323b9bd8117597c04570148dd294c5cb4040ad0db4f8
*PK*_RA_	a515e63088c264d95a8c91c74bdb6c077517e2808db16d12d5274af614057f3012701844def11f1b
*ID*_CS_	d1887486b3889ffabeae7c56da505a87603a1a74
*T*_1_	c5f2eaa6
*PID*_CS_	33b0fb30ae7fdc248e08b3205cc6435c6be9209e
*η*_1_	e6c1371fae23a212ba14dec04dc531a0cb9ed579a5921a4d32d4143e444ed6bdebaea3a23c624729
*P*_CS_	d8ade9b705ce815152e2445809ac1ded1f4134d2e62aafaccc96212cc581bc19bc330721ed0da3d2
ℂ_CS_	e0910483ba4e806d68fdcce29af792d31fdca35f0186652439b1d16a6605bdfd65503e6fc77d3d4c
*SK*_CS_	d86a63a66ad68bc2698cf69d02c5a375d6a119edef54b7a93c3737c65f297f45d00f3ec50 cd354cc
*PK*_CS_	3 cd47ce42038431eb8d8b1d7e525360d20a4cf093978ba9aef17f974872d5ca5920ddcce9e7133a3
*T*_2_	2eb2459a
*ID*_SM_	c652657799528d815d5e0dac700bf18ccb94e6ed
*PID*_SM_	33b0fb30ae7fdc248e08b3205cc6435c6be9209e
*TID*_SM_	7ad81c129b5fab92a7d280f79fefa72bcff1b0ec
*η*_2_	258eccd718b51e2832b914b00fab01486cb6f23a801b0d3aa1912e7fd3a11938cca5ddf77dc41b10
*P*_SM_	f12797dd1b62bc94b0e39800ddd2f45e86a754f8cfa433de08137da0a67333a459b91094c75db808
ℂ_SM_	fff557f20b23195267717feafcc728345d75b533d649d479278a3c6bc9390bbde754fb8b1908d37b
*SK*_SM_	c828b317824e25437ba8f0825cde831ac1698c60077437f8abe834d067b43d42e33c0150b728a810
*PK*_SM_	8686d1e33e0f87684345447b8b835e1fed84af045268f4822bbd9d0e81f325c9c120d5268d93f558
*T*_3_	da555fde
*ID*_SP_	f83549663bdb92cba628da33b7df6ed2da9ed81a
*PID*_SP_	f9b954a9d1822401fbe5444a53c1fd873ff54d10
*TID*_SP_	3d1e67fd3c86f793ae97ce05f6bb0baa0d24063b
*SK*_SP_	aa3e10c351da5b4e044d520f14102589918b3d22ce0915053294e28df56568f77416143d51a21480
*PK*_SP_	44cc49815f5d19fe74716abbdcc261bd60c48e71e64eca9ea6c00b87cbab333fe2dc3df99763a968
**ℝ**_1_	1844197e94aa53c1e665b61d085d8617059b0a8e
*T*_4_	90f98e57
*A*_1_	77ae2baa3c19d3942e2a0f4de4b69bb37ea00c6f
ℤ_1_	d50f3310c3ae1daed346cfcb5b9125a141d6e9b5
*T*_5_	fb3d199c
*T*_6_	844d1512
ℝ_2_	a2f43670749697dc6184f10e2cb71a44668c71cf
*A*_2_	1599a3ca42ce2a9ab41f308aa37b5df661610763
*A*_3_	9a11272d41d109bf67c5e3f1e3ea9ab83297caa9
*ɸ*_SM_	a490325f380261dac87dfa22e69f1f0be0605aa4
ℤ_2_	f970c169a3ab2bcf44f8d547d3563f6ebf96e016
*TID*_SP_^New^	cbebafaaf7acdd9e8e56207b5ac38d9b079ceb81
*TID*_SP_*	ea1a214e9dac82ca57f43f05dcc12021d320974c
*T*_7_	a288fee5
*A*_4_	71741324c7feb74445c94d73690ffc1da8087cf1
*ɸ*_SP_	eea8d4cdb368876e59a7aa11208b275af1cba489
**ℝ**_3_	17efd53cf1ce7f1897050303098b46f21e84bfc4
*T*_8_	e93b54d8
*B*_1_	e1b012d40fe67bc49b2752609f7b62e769077df0
*PID*_CS_*	c2777693c08db7b103757b303cca2feb51e23f38
ℤ_3_	87e92259194d88cdb03e3143097d25f11547d11f
*T*_9_	afe6ded0
**ℝ**_4_	548d809e4c0e4050e32c610d2bc6206fa3c82bf9
*T*_10_	c289d7be
*B*_2_	aba97cf8dfcdf19692beb3b1a67cdc9ba411af74
*B*_3_	6a37120bbbfba34968c02dcb5712ba3e154beff5
*ɸ*_SC_	b9449dd39a19ed0f8850116d65b047abdf67b7cf
ℤ_4_	fc5298c9db0f326ec3d95abc76fe7a3a1e625331
*TID*_SM_*	30120309f51969b22bb81079a668271596cdef99
^*T*11^	e76b09ca
*B*_4_	f838a873ff8fcb84c7e01d42a1a72cec63b9d697
*ɸ*_CM_	97996f0129d1f255be7924ecf70101114aee320a
*TID*_SM_^New^	989619de2bdb9f58ca8d9a2ba5711018abec3a0b
$E_{{sig}_{\beta_{n}}}$	ffc69a7d5a678427fad836a27d71d34c016e239b
ℍ_P_	147362e1d769d0df253f0310128684ae08692e79
ℍ_C_	4a24472953754a297d2772efde034898d23dc63e
${ID}_{C_{B}}$	11977c6bd7abf2c7107b1104c5e8e586e6672d7e
*Ƭ*_stp_	758e46c1
${SK}_{C_{B_{i}}}$	ffaf8f370d9747d790c9f1abd761e4f4e61710552922b55bea1804f2c9860ca3e9e07fdcf951634d
${PK}_{C_{B_{i}}}$	aae84e9c7bdb6820d49c5e810e6e4e442a8ed6f27275e7c9051e75a871aa2d8d8f79fb73fc929729
${\mathbb{Z}}_{V_{rs}}$	5913332a8dab2f31c7b7167a76056c937cd9b85b
$T_{C_{BR}}$	5e28fb8c
${ID}_{{SD}_{k}}$	315d3752b6cd74e5a2d24fc46fd5ec67a218ebd1
*T*_12_	9de92225
${PID}_{{SD}_{k}}$	55ce293c035e44134823fbbffd95bc9fe6088e96
${TID}_{{SD}_{k}}$	6b38b67d4487a108cee3aaf1181f372f176ab5bd
${SK}_{{SD}_{k}}$	96a51bb63b69ea5cec00acbeb62412f8015320499fb5c5e74f9ac4b5cf2ae4e5199dee2557d9fa7d
${PK}_{{SD}_{k}}$	d1bcddcebd75c13df5f8ee363ba2e62a6eeb4ced602957 cd39924738577ce5a3d17b820c1baf7c28
